# Characterizing Flocculated Mineral Sediments with Acoustic Backscatter, Using Solid and Hybrid Scattering Models

**DOI:** 10.1021/acs.iecr.3c01874

**Published:** 2023-09-27

**Authors:** Alastair S. Tonge, Jeffrey Peakall, Alexander P. G. Lockwood, Martyn Barnes, Timothy N. Hunter

**Affiliations:** † School of Chemical and Process Engineering, University of Leeds, Leeds LS2 9JT, U.K.; ‡ United Utilities Group PLC, Warrington WA5 3LP, U.K.; § School of Earth and Environment, University of Leeds, Leeds LS2 9JT, U.K.; ∥ Sellafield Ltd., Hinton House, Birchwood Park Ave, Warrington WA3 6GR, U.K.

## Abstract

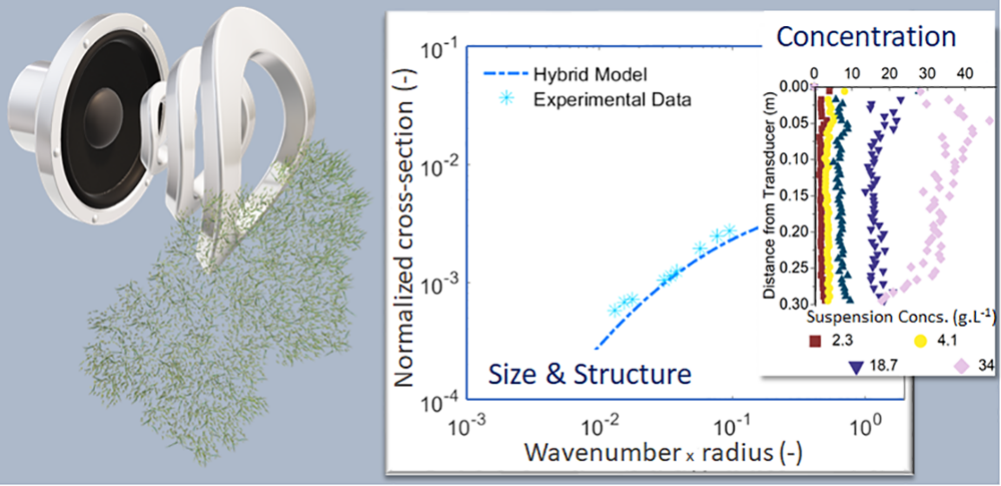

This study investigated the performance of an acoustic backscatter system (ABS) for the *in situ* particle characterization of complex wastes. Two sediments were used: a fine, milled calcite that was flocculated with anionic polyacrylamide and naturally flocculated pond sludge. Particles were initially measured independently by light-based techniques to gain size, the coefficient of variation (COV), and fractal dimensions. For acoustic experiments, a bespoke, high-fidelity ABS was employed with 1, 2.25, and 5 MHz probes and a recirculating mixing tank. Initially, the concentration independent attenuation and backscatter coefficients were measured for each system using a robust calibration procedure at multiple concentrations. Comparisons of the total scattering cross-section (χ) and form function (*f*) were made between the experimental data and two semiempirical models: a Solid Scattering model and a Hybrid model (where the effects of bound fluid are incorporated). Experimental data compared more closely to the Solid Scattering model, as it was assumed scattering was dominated by small, bound “flocculi” rather than the macroscopic structure. However, if the COV was used as a fit parameter, the hybrid model could give equally accurate fits for a range of input aggregate sizes, highlighting that important size and structure information can be gained from the acoustic models if there is some *a priori* system data. Additionally, dual-frequency inversions were undertaken to measure concentration profiles for various frequency pairs. Here, the lowest frequency pair gave the best performance (with accurate measurements in the range of 2–35 g·L^–1^) as interparticle scattering was lowest.

## 1 Introduction

There is currently a large drive to improve the cleanup of legacy nuclear waste deposits worldwide, enabling safe, long-term storage, and to allow the industry to better reposition itself as a crucial source of low-carbon energy for the future. For example, there are a variety of pond and silo sludges stored within the UK at the Sellafield Ltd. licensed site (one of the largest nuclear sites in Europe) which have formed complex suspensions with a variety of sizes and physical properties, containing both radiological and toxicological hazards.^1^ Knowledge of the settling and transport dynamics of these wastes would allow for optimization of thickening and pumping operations that are necessary for final abatement.^2,3^

The development of novel techniques to characterize the waste particle size and concentration in such hazardous environments is, therefore, imperative to allow for efficient and safe processing operations. Acoustic devices represent a promising technique, as they are used extensively to measure sediment transport in estuarine environments^4^ and, by appropriate adjustment of frequency range, size, and concentration, can also be utilized nondestructively and nonintrusively.^5,6^ In nuclear applications, ultrasonics have been used for *in situ* bed profiling^7,8^ and nondestructive testing^9^ but have not been applied for concentration measurements. More generally, ultrasonics are increasingly a critical technique for the in-process evaluation of multiphase mixing and transfer systems in a variety of industries.^10,11^

Acoustic devices for measuring particle size and concentration are generally used in either a forward transmission or a backscatter setup. In transmission, an ultrasonic signal is generated by one transmitter, and the signal is “caught” by a separate receiver. Acoustic transmission techniques for process monitoring have been studied by several groups as a method for particle measurement from the signal attenuation for many mineral and glass suspensions, for example.^12,13^ A transmission setup can also provide size measurement by measuring the frequency dependency of the backscatter strength, attenuation, and the “peak-frequency”, at which the backscattered power is greatest. Research into the development of theoretical equations governing these relationships and their experimental validation has been extensively undertaken to include factors such as morphological irregularities, temperature, and material properties.^12−15^

By operating in pulse-echo mode (i.e., a receiver angle of 0°), acoustic reflections from particles at multiple distance points in front of the transducer can be collected, and a distance profile of backscattered signal strength can then be produced. These are referred to commonly as acoustic backscatter systems (ABSs) and may be preferred, as a single transducer can be inserted *in situ* (or attached to the outside of pipelines) to reduce intrusion. The work of many authors^16−24^ has led to the development of equations to relate the backscattered voltage to particle concentration and mean size, so long as two key parameters that describe the scattering characteristics of the suspended particles are known; that is, the ensemble backscatter form function (*f*) and the scattering cross-section (χ). Physically, the form function is the ratio of the backscattered pressure to incident pressure (i.e., the relative scattering strength) for a 3D scatterer as a function of distance from the transducer. Conversely, χ is a measure of attenuation and increases as the backscattered pressure decreases, due to more energy being scattered away (or absorbed) from the sensor, as it quantifies scattering from a particle over all angles relative to its cross-sectional area. While these terms have been defined as a function of particle size and ultrasonic frequency for spherical beads and irregularly shaped sand particles,^16,24^ they have not been determined exactly for many cohesive/flocculated fine sediment systems, while modeling efforts for such complex suspensions have also been relatively limited.^23,25−27^

In terms of flocculated sediments in particular, the foundation work of Thorne et al.^23^ on backscatter modeling sought to incorporate the effect of fluid as the size increases and more water is incorporated into the particle structure. The interaction between the macroscopic aggregate and the microstructure is also critical, as considered in work by MacDonald and co-workers, who determined that scattering was often dominated by small “flocculi”.^26,27^ Recently, Pedocchi and Mosquera extended this theoretical approach,^28^ finding that coherence in the acoustic returns from primary particles within a floc may result in a much greater intensity than if particles were desegregated. Results also highlighted the influence of particle size distribution, as well as floc size on the backscatter, while the attenuation was dominated by the primary particles.^28^ Despite such progress, it is still largely unknown how widely applicable these fluid-particle models are to different flocculated sediments and what the best approaches are to extract structure parameters (such as floc polydispersity and fractal dimension) without other independent measurements.

Additionally, inversion methods are increasingly being applied to derive mean size or concentration profiles, both for complex sediments in estuarine environments^29−32^ and industrially,^6,33,34^ using either a single frequency^4^ or dual-frequency inversion methods.^20^ The advantage of multifrequency methods are that they can reduce numerical instabilities in the far field.^21,35^ However, a more complete understanding of the frequency ratio is required, and the concentration limitations of these inversions for flocculated systems are often unknown.

Therefore, this study aims to systematically assess the ability of acoustic backscatter to characterize flocculated mineral sediments. Specifically, both natural and engineered flocculated suspensions were chosen to represent nuclear simulant sludges. A bespoke, high-fidelity ABS was used across a frequency range of 1–5 MHz to gain measured backscatter and attenuation coefficients, to determine *f* and χ values. Then, both an irregular solid scattering model and the flocculated scattering model proposed by Thorne et al.^23^ were compared to the measured data for both systems, where model predictions were also fitted to improve estimates of polydispersity and size. Dual-frequency inversions were also performed by using different frequency pairs to gain concentration profiles, giving insight into the optimal frequency ratio and attenuation limits of the technique.

## 2 Acoustic Backscatter Theory

### 2.1 *G-*Function Modeling for Attenuation Determination

The fundamental equations of backscatter acoustics are described within the Supporting Information (SI, eqs S1–S3).^4,18,36^ Here, the backscatter voltage can be directly related to a number of particle parameters and acoustic system constants. Importantly, the scattering of any arbitrary particle system can be categorized based on the scattering (*k*_*s*_) and attenuation (α_*s*_) coefficients. A further transducer constant (*k*_*t*_) is used to normalize the electromechanical performance of specific transducers. The attenuation and scattering coefficients can be assessed on a dimensionless basis, through the form function (*f*) and scattering cross-section (χ), respectively (eqs S2 and S3).

In order to be able to experimentally determine the attenuation coefficient of suspensions, previous researchers^22,26,37,38^ have taken various approaches to linearizing the voltage (eq S1) with respect to distance, by taking the natural logarithm of the product of the measured voltage, *V*_*rms*_, such as given by *G* in eq 1.
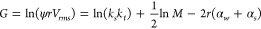
1If the particle concentration, *M*, does not change with distance from the transducer, *r*, the derivative with respect to *r* gives eq 2, with the requirement that such a relationship only holds for a homogeneously mixed system (scattering constant is not a function of distance).
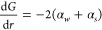
2Differentiating with respect to the mass concentration, *M*, produces eq 3, in terms of the mass independent attenuation coefficient, ξ, as proposed by Rice et al.^22^
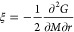
3Thus, by taking the gradient of *G* plotted against distance,  can be determined at multiple concentrations for a given particle system. Then,  can be plotted against concentration and the linear gradient used to find ξ.

A calibration following the *G-*function method is also given by Bux et al.^39^ and Tonge et al.^34^ for finding the transducer constant, *k*_*t*_, and the scattering constant, *k*_*s*_. Once the attenuation coefficient, ξ, is known, *k*_*t*_ can be estimated at known low or intermediate concentrations for well characterized monosized spherical particle systems via rearrangement of eq S1 into eq 4. First, values of the sediment backscatter constant, *k*_*s*_, must be estimated using a heuristic expression for disperse particles, such as that provided by Betteridge et al.^16^ or Thorne and Meral^24^ (see Section 2.3). As the transducer constant is sediment independent, it can then be used for all further studies with the same transducers.
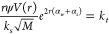
4With the transducer constant estimated, the backscattering constant can also then be measured experimentally for any system by simply rearranging eq 4, using the measured attenuation coefficients. This procedure was used herein to define the backscatter and attenuation coefficients experimentally for all of the investigated aggregated dispersions.

### 2.2 Solid Scattering and Hybrid Scattering Models

For irregular solid scatterers, such as noncohesive sediment encountered in fluvial environments, Thorne and Meral^24^ used experimental data from a number of authors to fit heuristic expressions for the form function, *f*, (eq 5)^37,40^ and the scattering cross-section, χ_*ss*_, (eq 6)^37,39,41^ as a function of *ka* (where *k* is the wavenumber and *a* is the particle radius). These equations allow the analytical modeling of the scattering response of solid particles (through *f*) and the attenuation (through χ) for any specific particle size, concentration, and transducer distance. However, it is important to note that it assumes relatively dilute conditions, where interparticle scattering does not occur. As the unflocculated sediment used in this study was in a low *ka* range, viscous attenuation was also considered, as given in Urick’s model,^15^ and expressed in terms of the viscous dimensionless χ_sv_ function, detailed within the SI (Section S2, eqs S4–S7). Therefore, total attenuation was modeled for solid scatters (χ = χ_ss_ + χ_sv_).
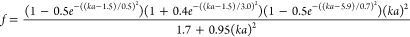
5
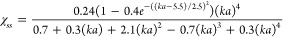
6However, the above “Solid Scattering” model takes no account of the unique properties of “flocs” (large aggregates formed by polymer flocculation), in terms of the interaction of sound with the bound fluid. In order to model flocculated particle acoustic parameters from unflocculated primary particles to large, low-density flocs, Thorne et al.^23^ detailed a “Hybrid” model to take account of both fluid and solid properties. First, they expressed the density and compressional wave speed of the scatterers as a function of the object size. The density of the floc as a function of size, ρ_*f*_(*a*), is given in eq 7.
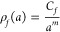
7Here, *C*_*f*_ (kg·m^(3-m)^) and *m* vary depending on the process of flocculation.^42^ The parameter *C*_*f*_ is an empirical fit to combined size and settling data^43^ and captures the density of the sediment and the primary particle size, while *m* is a measure of the fractal dimension. Once the floc density is known, the density ratio (or specific gravity) between the particle and fluid can be found (γ, where γ *= ρ*_*f*_*/ρ*_*w*_). It additionally allows calculation of the porosity of the floc (ε, where ε = *(ρ*_*s*_*– ρ*_*f*_*)/(ρ*_*s*_*– ρ*_*w*_*)*). Following this, the ratio of the sound velocity in the scatterers to that in the fluid, ζ(*a*), can be defined using Wood’s^44^ equation, by assuming the solid and water components contribute to the bulk compressibility in proportion to the porosity of the particle, ε,^23^ as in eq 8.
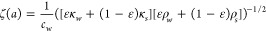
8Here κ*_s_,* κ*_w_*, ρ*_s_*, and ρ_*w*_ are the compressibility and density of the sediment and water, and *c*_*w*_ is the speed of sound in water. For both the sediment and water, it is assumed that the compressibility is given by *κ* = 1/*ρc*^2^. While this assumption is not technically correct for the solid primary particles, it causes ζ(*a*) to approach the correct value as the porosity approaches zero and results in similar predictions between the Hybrid model and the Solid Scattering model for primary particulates. When performing model calculations, the maximum density is set to be that of the solid primary particulates and the minimum density was set at 1020 kg·m^–3^ (as was used by Thorne et al.^23^).

Having defined the sound speed ratio and density of the particle as a function of floc size, a modified form function, *f*, (eq 11) and the scattering cross-section, χ, (eq 12) can then be calculated by using expressions from Medwin and Clay^45^ (originally given by Johnson^46^) to find the corresponding constants, *k*_*fα*_ and *k*_*ff*_, (eqs 9 and 10). These represent the change in floc acoustic scattering and attenuation parameters with sediment density, compressibility, porosity, and compressive wave speed under the assumption that the flocs act as fluid scatterers. The constants are subsequently used in heuristic expressions for χ and *f*, that are otherwise a function of *ka*. The subscript “*fi*” indicates an irregular fluid sphere.
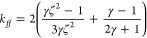
9
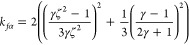
10
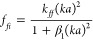
11
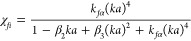
12The values used for the coefficients β_1_, β_2_, and β_3_ by Thorne et al.^23^ were 1.2, 1.0, and 1.5, respectively (noting they were listed as epsilon, ε_*1–3*_, in the original study); but it was stated that these values may depend on floc structure, and their variability is still to be understood. Coefficients were determined by fitting the produced heuristic form function and scattering cross-section to the fluid sphere model from Anderson^47^ between *ka* = 0.2 and *ka* = 2. The form of the heuristic expressions used is similar to that of the “Solid Scattering” model (eqs 5 and 6). The Hybrid model thus represents the solid particle scattering characteristics for small particle sizes and transitions toward modeling a fluid sphere as the size increases and more water is incorporated into the structure of the modeled floc.

### 2.3 Dual-Frequency Inversion

A critical industrial function of acoustic backscatter systems is their use as concentration profilers. Here, a dual-frequency inversion approach was used, as described by Rice et al.,^22^ from models proposed by Bricault^48^ and Hurther et al.,^20^ shown in eqs 13–15. Eq 13 represents the squared form of the voltage return simplified to two terms: *J(r)* and Φ^2^(*r*). The *J(r*) term (eq 14) contains the sediment attenuation coefficient, ξ, and mass concentration, *M*, while Φ^2^(*r*) (eq 15) contains the sediment backscatter and system gain constants, *k*_*s*_ and *k*_*t*_, the attenuation due to water, α_*w*_, and the near-field correction factor, ψ.
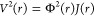
13
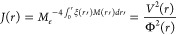
14
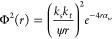
15

If the object size and, therefore, ξ and *k*_*s*_ do not change with distance from the probe, as would be the case for homogeneously mixed suspensions, then the attenuation term can be moved outside of the integral and written as
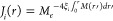
16where *i* = 1, 2 for two different frequencies 1 and 2. Taking the natural logarithm, dividing by ξ_*i*_, and rearranging for *M* gives eq 17.
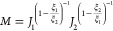
17

For this method, the attenuation ratio ξ_1_/ξ_2_ must be sufficiently different from unity to prevent mathematical instabilities and subsequent errors. However, the optimal frequency pairing has not been comprehensively investigated. Previous work by the current authors considered pairings of 2–2.5 MHz (from single broadband transducers).^34^ Here, a wider range of frequency pairings is investigated, for three probes with central frequencies of 1, 2.25, and 5 MHz.

## 3 Materials and Methods

### 3.1 Materials

Two aggregated and flocculated sediments were used in this study. The main test material was fine, milled calcium carbonate (calcite type) Omyacarb 2 (Omya UK Ltd.). The particles have been previously characterized as being slightly cationically charged at neutral pH, with a degree of natural aggregation^49^ from the low surface charge. Polymeric flocculation of the sediment was achieved with an anionic high molecular weight, medium charge density polymer AN934SH (SNF Ltd., UK) chosen to induce bridging flocculation.^50,51^ Previous studies have investigated very similar flocculated calcite systems, largely for mineral processing.^2,52,53^

Second, natural sediment from the base of a dairy farm pond (in Appleby-in-Westmorland, UK), obtained by Barrnon Ltd. UK, was selected to represent naturally occurring flocs similar to wastewater sludges and some nuclear wastes.^54^ Referred to as Barrnon Pond Sludge (BPS) hereafter, it has recently been extensively characterized, comprising of high organic content (>50%) and mixtures of silts, clays, and diatoms, with the sediment being overall negatively charged.^55^ The BPS also showed good levels of natural flocculation from existing biological polymeric material, without the addition of synthetic flocculation agents.^55^ Additionally, spherical glass powder, Honite 16 (Guyson Ltd., UK), with a median size (*d*_50_) of 78 μm and a low coefficient of variation (COV) of 0.21, was initially used to calibrate the acoustic transducer coefficients (*k*_*t*_), as utilized previously by the current authors.^34^

### 3.2 Flocculation and Aggregate Size Characterization

Flocculation of the calcite was conducted in the same calibration tank as used for the acoustic analysis (Section 3.3) and as described previously by the current authors.^34^ Briefly, it was an impeller-agitated, 0.8 m tall by 0.3 m diameter column with a 0.2 m conical base and an outlet for recirculation. The column was fitted with 4 × 0.02 m baffles to reduce vortex formation and a 0.08 m diameter 45° axial flow impeller. A peristaltic pump (Watson Marlow 520R), running at the maximum flow rate of 200 rpm with 3/4 in. diameter tubing, was used to redistribute suspension from the conical base to a manifold arranged at the top of the tank, preventing particles from settling out and to ensure mixing homogeneity.

Suspensions of the calcite powder were initially mixed in the column at a concentration of ∼40 g·L^–1^, with a 40 ppm dose of the polymer slowly added dropwise over 1 min (from an initial stock solution of 1000 ppm). This dose has previously been found to give optimal flocculation and settling properties of the same calcite-polymer system.^2^ While this concentration is much greater than typical in mineral processing operations,^56^ where polymer dose is industrially limited, the objective here was to maximize the stability of the flocs for the acoustic calibrations. Mixing was conducted with the impeller at 450 rpm initially and dropped down to 150 rpm after 15 min, where it was assumed the flocs would remain relatively consistent in size from the lower shear rate.^2^ It was anticipated that a considerable degree of shear degradation and densification may occur over the initial high-shear mixing (where fines can be broken and recombined). However, previous work on similar flocculated mineral systems generated aggregates that were relatively highly stable for analysis, while still demonstrating significant differences to the nonflocculated constituent particles.^57^ Similar concentration and mixing settings were used to make suspensions of BPS for analysis, although no flocculant was added.

Sizes for mixed flocculated and unflocculated calcite, as well as the BPS, were measured using a Mastersizer 2000T (Malvern Panalytical Ltd., UK). Aliquots of flocculated particle samples obtained during the initial mixing regime described above were then taken to the Mastersizer cell and placed under the correct obscuration. Shear in the Mastersizer was controlled by minimizing the mixing rate on the attached dispersion unit (Hydro 2000SM) while still maintaining a valid measurement signal (typically ∼1500 rpm). It is noted that the cell did, therefore, impart additional shear that may have led to some partial breakdown of the flocs. Further, raw intensity-angle data collected in the Mastersizer allowed for measurement of the fractal dimension, using the method employed by Zhou and Franks^50^ as also detailed in previous investigations.^55,57^ Fractal dimension (*D*_*f*_) fits were taken over 20 angles with a search algorithm employed using Matlab 2019b (The MathWorks, Inc.) to find the range over which the correlation coefficient was greatest.

Size distributions for the particulate and flocculated calcite, as well as the BPS, are shown within the SI (Figures S1 and S2, respectively) in terms of given volume% and translated number% distributions (the latter being used for the acoustic modeling). Scattering *log[I(q)]* versus *log[q]*, and resulting linear fits for *D*_*f*_ calculations, are also given within the SI (Figure S3). A full summary of the particle characterization data is given in Table 1.

**Table 1:** Measured Solid Properties of the Calcite Particles and Flocculated Aggregates (flocs) and the Barrnon Pond Sludge (BPS)

**Material Name**	**Mean number radius, a**_**0**_**(μm)**	**Median volume diameter, d**_**50**_**(μm)**	**Coefficient of Variation,(COV)**	**Fractal Dimension**	**Particulate Density(kg·m**^**-3**^**)**	**Floc Density(kg·m**^**-3**^**)**
Calcite (particles)	0.7	4.3	0.70		2710	
Calcite (flocs)	14.5	107.6	0.75	2.35	2710	1238
BPS	0.92	21.4	0.78	2.12	2650	1525

The nonflocculated calcite (particulates) represents a moderately broad size distribution (coefficient of variation, COV = 0.7) with a small degree of aggregation present indicated by the minor peak at 100 μm (consistent with previous work^49^) although, these aggregates are negligible on a number basis. After flocculation, a distinct size increase is evidenced, accompanied by a slight growth in the COV to 0.75. The change in the COV is likely caused by some degree of floc breakup over time. The BPS particulates have a naturally wider distribution (which may be expected, given its heterogeneity) with a mean peak at ∼20 μm and a further large peak at ∼700 μm. Previous investigations of this material have shown the BPS to be clusters of much smaller micron and submicron diatoms and platelets.^55^ Indeed, on a number basis, the mean particle radius of the BPS used for acoustics modeling (*a*_0_) is only 0.92 μm, and so not much larger than the particulate calcite (*a*_0_ = 0.7 μm).

Fractal dimension values correspond to those typically found for flocculated mineral and natural sediments (*D*_*f*_ = 2–2.7)^26,50,55,57^ indicating relatively porous structures, as opposed to that of a solid sphere (*D*_*f*_ = 3). While the known density for crystalline calcite was used (2710 kg·m^–3^) BPS sludge particulate density was estimated to be the same as that of kaolinite (2650 kg·m^–3^), as used by Thorne et al.^23^ for naturally occurring marine flocs. Additionally, it has been found by other authors that the soil primary particle density is typically in a range of 2500–2700 kg·m^–3^ for mineral soils.^58^ A high organic matter content in the BPS may, however, lead to reduced density compared to more mineral rich soils. The overall floc density was calculated from the measured fractal dimensions and median sizes.^55,57^ The BPS is overall a denser floc, despite the lower fractal dimension (indicating a more open internal structure), due to its smaller average size.

### 3.3 Acoustic Calibration and Analysis Methodology

Acoustic calibrations (used to gain experimental attenuation, ξ, and scattering coefficients, *k*_*s*_, for the suspensions) were undertaken in the same calibration column as that discussed for the sediment flocculation. Measurements were conducted with a bespoke high fidelity acoustic backscatter system (ABS), the Ultrasound Array Research Platform (UARP Mark II-16), featuring 16 individual transducer connections, as described in a previous publication.^34^ For testing, three transducers (PIM501, PIM5025, and PIM3750 from Sonatest Ltd., UK) were arranged in an equilateral triangle, facing vertically down in the tank, about 35 cm above the impeller (and fully immersed). The transducers have central frequencies of 1, 2.25, and 5 MHz and were additionally pulsed at ±15% of the central frequency by the UARP. Backscatter voltage was gained in a distance region of 0–0.3 m (and so above the direct impeller, but within a region that was assumed to have consistent mixing). In experiments, the received echo voltage was recorded using 31172 points spaced over the 0.3 m range, with 10,000 repeat measurements made over a 5 min period.

Initially, the Honite 16 glass particle dispersions were tested at 2.5 and 5 g·L^–1^, to enable determination of the transducer coefficients (*k*_*t*_)^34^ (see SI, Figure S4). For the cohesive sediment studies, five nominal particle concentrations for calcite (both particulate and flocculated sediments) and four concentrations for BPS ranging from 2.5 to 35 g·L^–1^ were used. Importantly for the flocculated calcite, initially the highest (35 g·L^–1^) concentration was prepared (as outlined in Section 3.1). The dispersion was then diluted in stages using water with residual polymer so as to maintain the initial aggregate conditions. Samples were taken within the main measurement zone (0–0.3 m) before and after the tests for each system (see SI, Figures S5–S7 and Table S1) with no differences with height evident, and only some minor losses from sediment accumulating in the recirculating tubing during testing.

**Figure 1:**
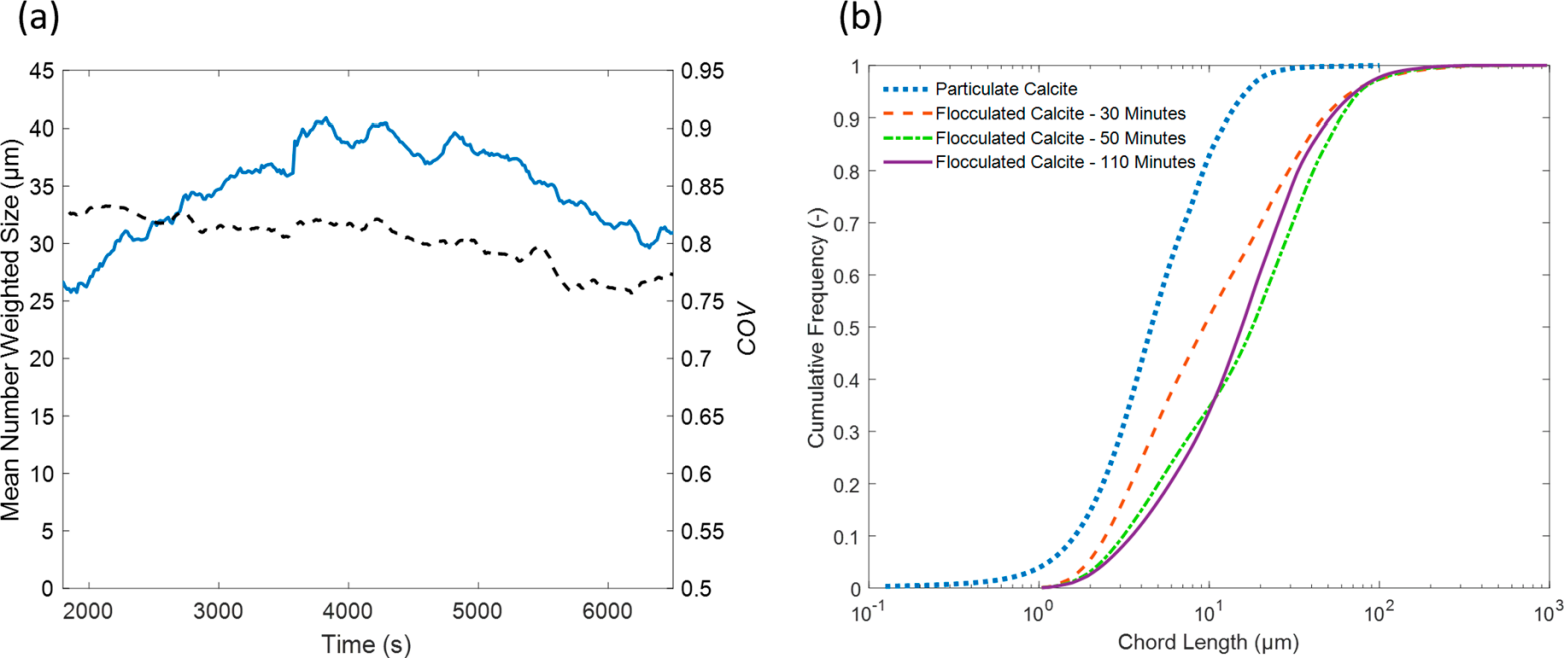
(a) Mean number diameter (−) and the COV (- - -) as a function of time produced by conversion of focused beam reflectance measurement (FBRM) chord length distributions. Suspension diluted in stages from 34.9 g·L^–1^ to 2.3 g·L^–1^ throughout the period shown. (b) Cumulative Frequency (as probability) chord length distributions of particulate calcite and flocculated calcite at the indicated times during acoustic calibration trials.

To further confirm that aggregate sizes were consistent, a focused beam reflectance measurement (FBRM) model D600S (Mettler-Toledo, UK) was used to take *in situ* measurements during the acoustics calibration (see Figure 1 (a) and (b)). Here, the probe was inserted at an angle of ∼40° to the vertical within the upper section of the column suspensions, approximately 25 cm from the impeller. The mean particle number count and coefficient of variation (COV) were determined from the raw chord length distributions, using the method proposed by Li and Wilkinson,^59^ as implemented by Johnson et al.^60^ The aggregate mean size (Figure 1 (a)) is fairly consistent at 35 μm ± 5 μm (noting acoustics measurements started at ∼2500 s, and so after the initial flocculation was equilibrated, and mixing shear reduced) with the COV also relatively invariant and similar to that measured by the Mastersizer. The example extracted raw chord length distributions (Figure 1 (b)) do suggest some degree of fines recombination may be occurring during the dilutions and extended mixing (with median sizes increasing slightly between the 30- and 50-min profiles). Nevertheless, total size distributions are very similar for all times, with differences to the nonflocculated calcite broadly maintained. This result gives confidence that the dilution procedure and time taken to completely perform the measurements did not lead to significant changes in the properties of the flocs. Also, there is good consistency in size data between the number-based distributions from light scattering for the flocculated calcite (Table 1) and the FBRM (noting that the *a*_0_ value in Table 1 represents the radius not diameter).

The Hybrid model^23^ was implemented in Matlab, with modifications made to implement the fractal dimension relationship to the primary particle size. The Hybrid model was chosen as a comparison to the irregular Solid Scattering model, as it incorporates the floc density and compressional wave speed that could be expected to influence the scattering and attenuation parameters of the flocculated particles, and has been shown to allow for more accurate modeling.^23,61^ The compressional wave speeds used in the Hybrid model were 5450 ms^–1^ for calcite, as an estimate from data collected by Verwer et al.^62^ For BPS, a value of 1400 ms^–1^ was used, as taken from Marshall and Lineback^63^ from sediment cored from Lake Michigan, as an estimate for typical sediment sound speed in lentic environments.

## 4 Results and Discussion

### 4.1 Determination of Acoustic Attenuation Coefficients

**Figure 2:**
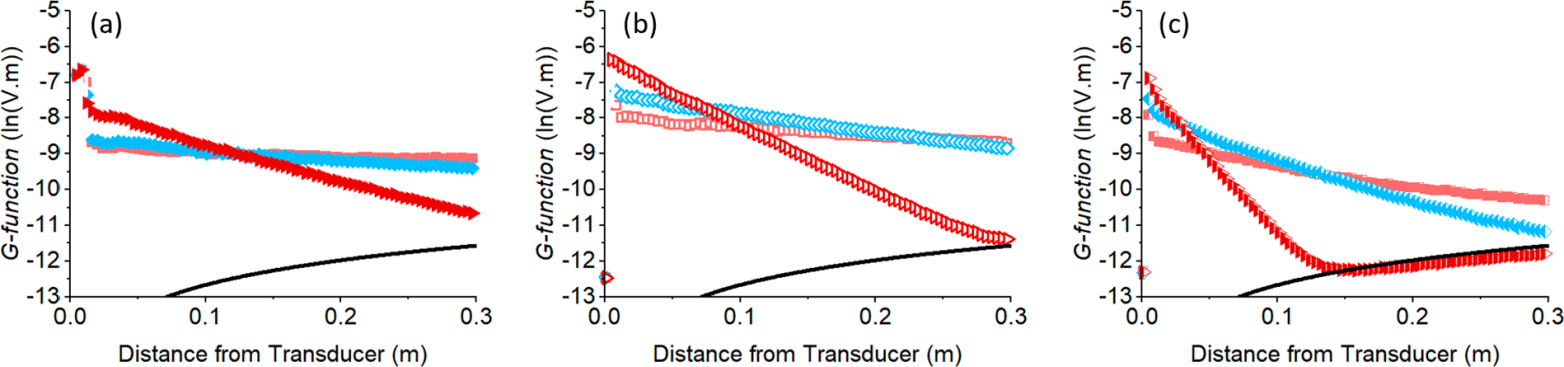
Measured *G-*function profiles ((a)–(c)) for flocculated calcite pulsed at 1, 2.25, and 5 MHz, respectively, from three particle concentrations (■ = 2.3 g·L^–1^, ◆ = 8.3 g·L^–1^, ▶ = 34.9 g·L^–1^).

**Figure 3:**
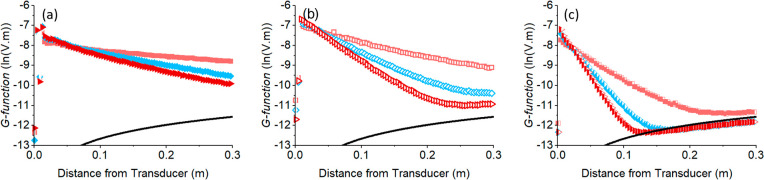
Measured *G-*function profiles ((a)–(c)) for BPS pulsed at 1, 2.25, and 5 MHz, respectively, from three particle concentrations (■ = 6.9 g·L^–1^, ◆ = 16.7 g·L^–1^, ▶ = 22.9 g·L^–1^).

Figure 2 presents the measured *G*-function profile with distance for the flocculated calcite at three concentrations, using the three frequency probes at their central frequencies (1, 2.25, and 5 MHz) with the BPS data shown for comparison in Figure 3. Results for particulate calcite are given within the SI (Figure S8). In general, profiles for both species are typical for moderate to strongly attenuating species (depending on frequency)^4,19,39^ with the expected linear relationship between *G-*function and distance, until the signal reaches the instrument noise floor (shown by the black line in the figures). The level of attenuation clearly increases with frequency (indicated by the gradient in *G* versus distance, d*G*/d*r*), and at higher frequencies and concentrations, the signal only penetrates moderate depths (∼0.1 m) until reaching the noise floor.

Interestingly, the *G*-function linearization has some variance in the BPS data at the lowest frequency (where the backscatter is much stronger than the attenuation). Given this is not from depthwise variation in the sediment, it may be from the innate heterogeneity of the sediment leading to addition complexities or multiple scattering effects enhancing the effective noise floor of the system.^35^ Nonetheless, the general consistency in data between the particulate systems gives confidence in the ability of the UARP to qualitatively measure the concentration and flocculation state of sediments with varying mineralogical and organic composition.

**Figure 4:**
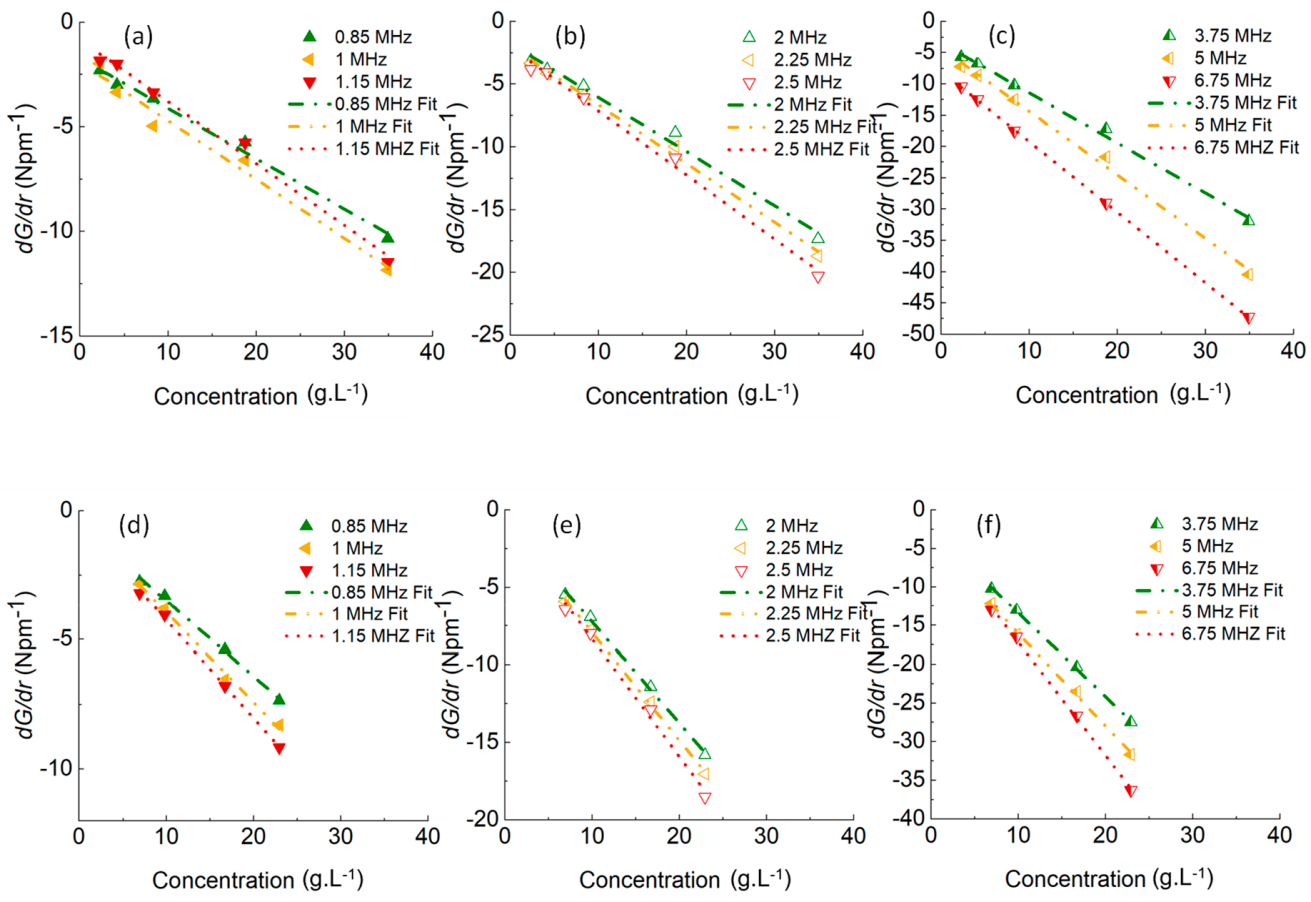
Gradient d*G/*d*r* versus concentration calibrations for flocculated calcite ((a)–(c)) and BPS ((d)–(f)) to allow for determination of attenuation coefficients at 1, 2.25, and 5 MHz.

The gradient of *G* versus distance (d*G*/d*r*) was extracted at intermediate distances (0.5–0.15 m) to enable the determination of the sediment attenuation coefficients (ζ) from d*G*/d*r* versus *M*, as summarized in Section 2.1.^22,34^ For higher attenuating systems that encountered the noise floor within this distance range (e.g., certain 5 MHz data), a smaller distance of 0.05 to 0.1 m was used to estimate the gradient. Figure 4 presents these concentration calibrations for flocculated calcite and BPS at all frequencies.

It is evident that within the concentration ranges studied, good linearity is observed in the data between d*G*/d*r* and concentration (which would be expected for well-mixed suspensions below levels of very high interparticle scattering^22^). The strong linearity of the concentration data also highlights that the extended mixing during measurements did not significantly alter aggregate sizes (especially in the case of flocculated calcite). The consistency in the data therefore gives confidence in the determined attenuation coefficients (taken as the −0.5 × average gradient). A considerable increase in attenuation is measured at the higher frequencies, as a result of the expected increase in scattering cross-section.^23,24,26^ Additionally, there is clear delineation in the attenuation gradients between each frequency pulsed from individual probes, leading to a large array of frequency pairs that may be used for dual-frequency concentration inversions.

Interestingly, the gradient attenuation is noticeably larger for the BPS than that for the flocculated calcite (which is the main reason measurements were taken over a slightly smaller concentration range). While the flocculated calcite is larger and therefore will be subject to greater scattering attenuation, the BPS flocs will likely have higher levels of viscous attenuation, which appears to dominate. Similarly, the particulate calcite also has slightly greater attenuation than the flocs (see SI, Figure S9). Changes within the aggregate microstructures may also play a role in the enhanced attenuation of the BPS. It is also emphasized that even if precise concentration profiles cannot be produced, the relationship between d*G*/d*r* and concentration could be used as a qualitative *in situ* concentration calibration, which would still be of great use for optimizing many industrial applications with requirements for remote measurements.

The specific concentration independent attenuation values (ξ) were extracted from these plots and are given within the SI (Table S2 for all frequencies). Using the defined transducer coefficients (Figure S4), the scattering coefficients (*k*_*s*_) were also determined for both the particulate and flocculated calcite, as well as the BPS suspensions, and are also given within the SI (Table S3). It is noted that the *k*_*s*_ values were calculated individually for each specific concentration, and an average value was used in the modeling analysis that follows (Section 4.2).

### 4.2 Comparison of Solid Scattering and Hybrid Models

In order to investigate the change in attenuation and backscatter strength more completely, the sediment attenuation coefficients, ξ, and backscatter constants, *k*_*s*_, were converted to their dimensionless equivalents, the scattering cross-section, χ (eq S3), and form function, *f* (eq S2), and density normalized through the specific gravity, as per the method of Bux et al.^39^ The density of the flocs was calculated from the mean size and fractal dimension values (see Table 1). Results are then compared to the specific gravity normalized Solid Scattering model^24^ and Hybrid Model^23^ using the measured coefficient of variation (COV) from the Mastersizer PSDs. Following this, an investigation into the variation of modeling results with differences in fractal dimension and the COV was undertaken. Specific gravity normalization has been shown previously to allow for comparison between data sets comprising varying particle densities.^39,64^

**Figure 5:**
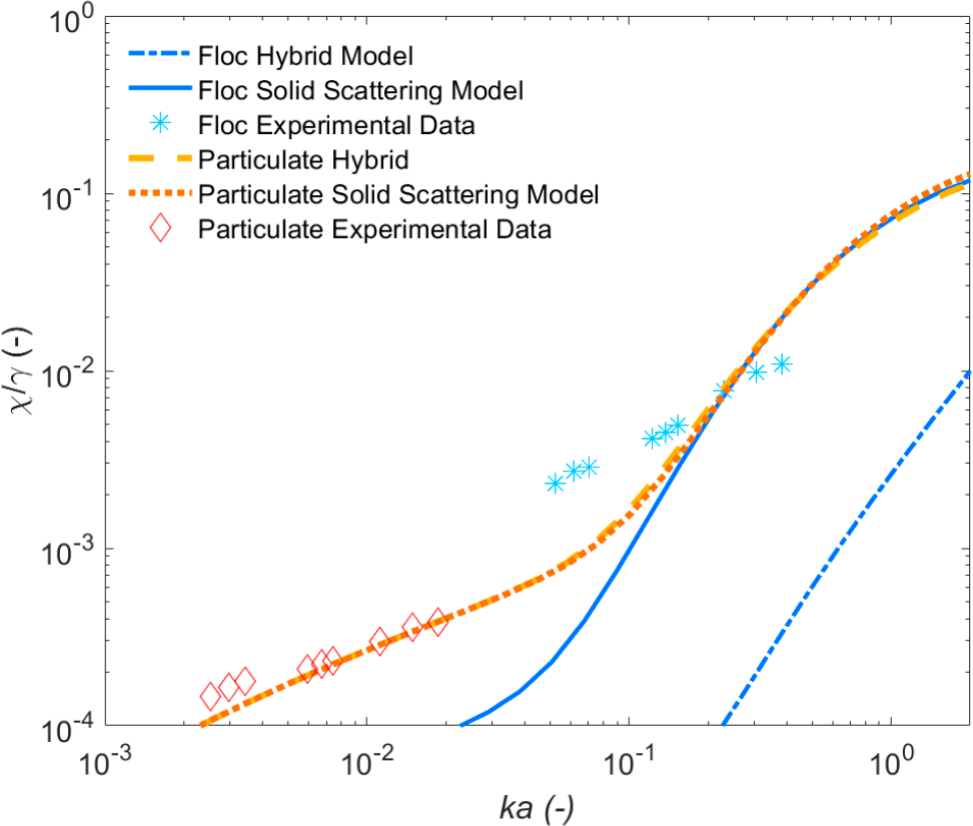
Specific gravity normalized scattering cross-section data and model fits for calcite flocs (*γ* = 1.24) and particulates (*γ* = 2.71), as a function of frequency expressed in terms of *ka*.

Specific gravity normalized cross-section versus *ka* for the calcite floc (blue) and particulate (red) calcite experimental data, as well as model fits, are presented in Figure 5 (using measured size, COV, and *D*_*f*_ data for both the experimental points and as inputs to the models).

For the particulate case, the specific gravity normalized scattering cross-section is seen to be in good agreement with model results, and bearing the expected trend with frequency typical of the viscous scattering regime.^39^ For the flocculated case, a decreased sensitivity to frequency is observed compared to both the Hybrid and Solid Scattering models, which could be attributed to a wider size distribution *in situ* than was measured. As similar COV results were observed by both the *in situ* FBRM and *ex situ* laser diffraction, this may be unlikely, although deviations between ABS and light-based measurements have been noted previously^27,65^ and possibly may be the result of the measurement limitation of the FBRM. Furthermore, the values for the model parameters (β_1_, β_2_ and β_3_, eqs 11 and 12) were the same as those used by Thorne et al.,^23^ where the dependence of these values on floc structure is still to be determined.

Most interestingly for the flocculated case, the experimental data are observed to be much more highly attenuating than estimated by the Hybrid model. The Hybrid model predicts a relatively weak attenuation response largely because of the low floc density (1238 kg·m^–3^) and hence low acoustic contrast (eq 8). The acoustic contrast value was used to calculate the irregular fluid form function and scattering cross-section and the resultant value normalized by the specific gravity of the floc. Thus, effectively, the flocs present higher levels of attenuation than that predicted from their open structure. To underline this, the Hybrid model was altered, by using different input fractal dimension values (*D*_*f*_ = 2.2–3), as shown within the SI (Figure S10). Increasing fractal dimension moves the prediction towards the experimental data and Solid Scattering model. However, floc data for the low *ka* range are greater than even predicted by the Solid Scattering model, suggesting a more complex attenuation interaction or potential errors from size or COV measurements.

A more accurate fit by the Solid Scattering model may not be entirely unexpected, as it has been proposed by Vincent and MacDonald^27^ that the acoustic signal is dominated by the scattering from smaller, tightly bound aggregates that make up the macro structure of the floc. In their study, these small microfloc aggregates (termed “flocculi”) were best modeled using the density of the unflocculated sediment, due to their small size and likely higher fractal dimension that are therefore more accurately represented in these results by the Solid Scattering model. Although the volume of large (∼100 μm) flocs was shown to be significant for the flocculated calcite used, their corresponding number count is low, with a number mean diameter of ∼30 μm. It should be noted that the experimental results presented here are also normalized with respect to the specific gravity and thus inherently take the floc density into account. The hybrid model also accounts for the change in sound speed in the floc with changing porosity/density. It cannot be stated with certainty whether the smaller flocs are denser and thus are better reflected by the Solid Scattering model or whether the Hybrid model has inaccuracies in determining the effect of porosity on the speed of sound in the floc.

**Figure 6:**
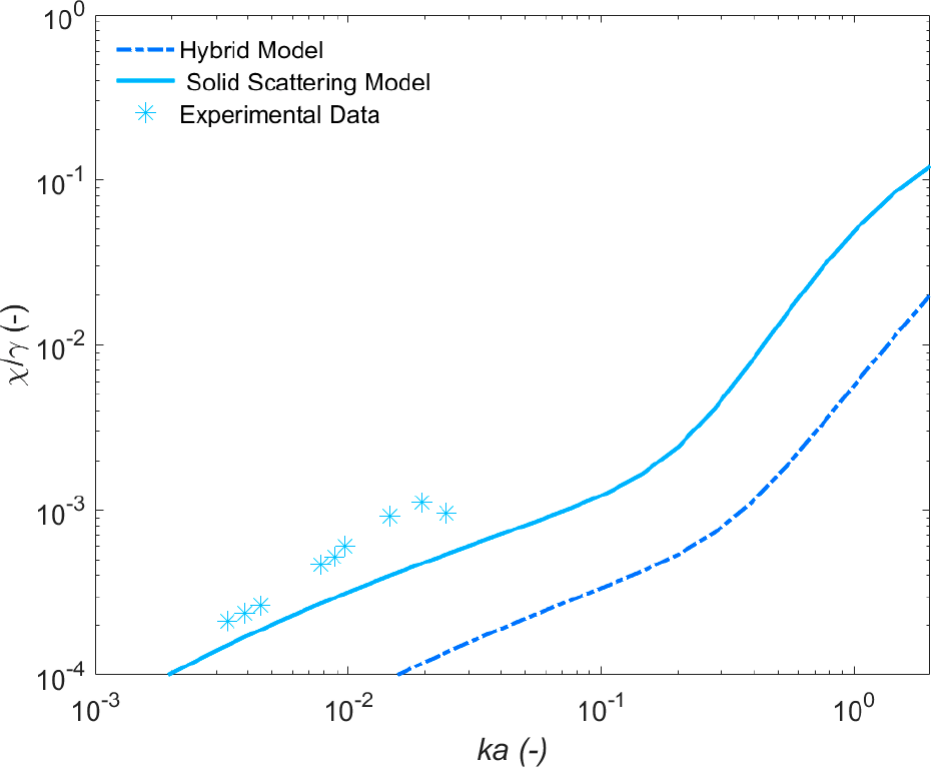
Specific gravity normalized scattering cross-section data and model fits for BPS, as a function of frequency expressed in terms of *ka*.

Normalized cross-sectional model comparisons for the BPS data are shown in Figure 6, again using particle properties determined from light scattering measurements. There is a reasonable fit to the experimental data by the density normalized solid scattering model, although a slightly higher sensitivity and larger scattering cross-section values in general. While the volume distributions for BPS exhibit a large degree of multimodality, the number distribution produced only a narrow peak at ∼0.9 μm with relatively low polydispersity indicated by the COV value (Table 1). An increase in both absolute values and frequency sensitivity of the model could also be achieved by a decrease in the COV, suggesting that perhaps only a narrow distribution of particles around the mean number value are dominating the acoustic signal. Indeed, if the input COV is reduced, differences between the data and the Solid Scattering model can be reduced toward parity (see SI, Figure S11).

Despite a good fit to the Solid Scattering model, the Hybrid model again underestimates the experimental attenuation values due to the low aggregate density (1525 kg·m^–3^) predicted from the measured floc size and fractal dimension. This underestimation of the density normalized scattering cross-section was also observed in the flocculated calcite results (Figure 5) although this effect is less evident in the case of BPS, because the much smaller number mean diameter results in a relatively higher floc density. The accuracy of the Solid Scattering model compared to the Hybrid model also supports the hypothesis of Vincent and MacDonald^27^ and Pedocchi and Mosquera^28^ that acoustic attenuation is due largely to the dense particulate clusters that are the building blocks of the overall floc structure. To further probe the model parameters, the effect of changing the compressional wave speed on the Hybrid model was also investigated (see SI, Figure S12). However, very little difference is evident between inputs of 700–2800 m·s^–1^.

Results highlight, in general, the importance of determining the primary particle size distribution, as this has been demonstrated to strongly affect the acoustic response across all modeled *ka* values. Interestingly, the compressional wave speed has a comparatively small effect at low *ka* but becomes more significant at high *ka*. For an induced change in the input compressional wave speed (+100% and −50%), the resultant difference in the estimated specific gravity normalized cross-section is relatively small for *ka* < 0.4. This indicates some degree of robustness of the model with respect to the wave speed, and that even if there are errors in the estimated speed used for BPS (1400 m·s^–1^^63^) it does not cause considerable model deviation from experimental results. While some degree of error likely exists in the input density to the model (as natural sediments will contain a wide variety of materials with differing densities quoted), densities from the literature indicate a relatively narrow range of around 2500–2700 kg·m^–3^^58^ and are therefore not thought to be the cause for the model deviation observed for the BPS data.

In terms of particle characterization, size and COV measurements taken from the Mastersizer are likely the largest sources of error, due to the shear that is inherent in the agitation system used to suspend the particles in the measurement cell. Excessive shear may cause aggregate breakup that would lead to a decrease in the measured size and differences in the COV. To illustrate the importance of accurate size and COV estimations, it was therefore decided to attempt to fit the Hybrid model to the experimental attenuation values of the flocculated calcite by purposely varying the input floc size over a specific range for the model (rather than using the light scattering value). Here, set floc sizes were used, and the data were fitted using the COV and fractal dimensions as initial free parameters. However, it was found that a fractal dimension value of 2.9 was an optimal fit in all cases (implying that smaller more compact flocculi dominate the attenuation); thus, only the COV was used as the variable parameter. Such a high *D*_*f*_ value may also indicate some errors with their estimation from static light scattering. Nevertheless, it is also known that small flocculi or microflocs are essentially nonfractal in nature, due to the scaling requirements between their constituent sizes and the larger macroflocs.^66^

**Figure 7:**
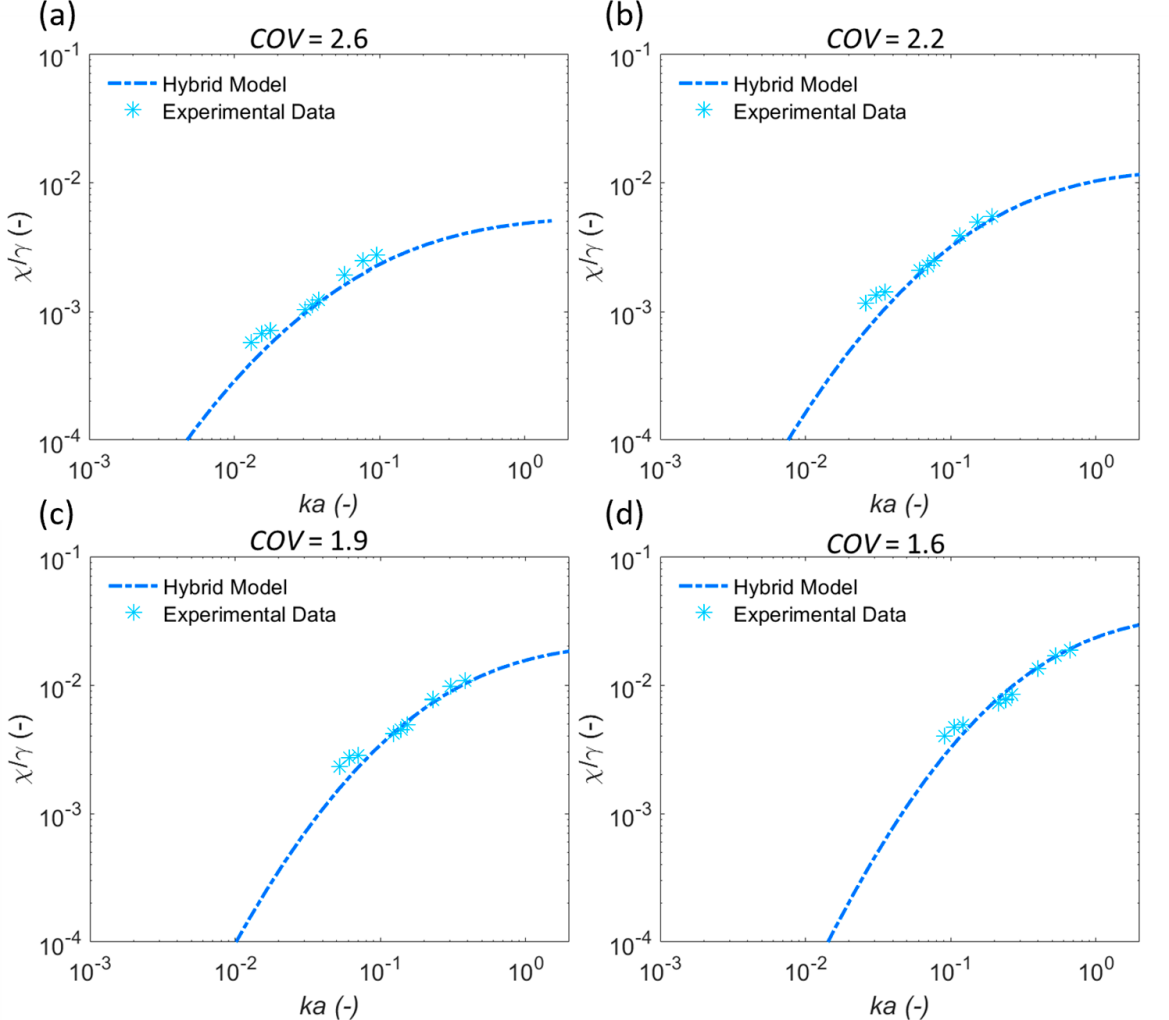
Fitted Hybrid model solutions to experimental data for calcite flocs at number mean sizes of a) 7.2, b) 14.5, c) 28.9, and d) 50, with corresponding floc densities of 2531, 2281, 2195, and 2131 kg·m^–3^ and fitted COVs, respectively.

Results from varying model floc sizes from 7.2 to 50 μm are given in Figure 7 (a)–(d) for the flocculated calcite, along with the fitted COV values and corresponding floc densities in each case. They indicate that if the floc PSD is unknown, multiple values of floc size can provide a good model fit by varying the COV used in the Hybrid model, although the COV reduces as the floc size increases. This result critically emphasizes the need to independently measure aggregate size or polydispersity accurately and in so doing signals a limitation of the acoustic backscatter models. Model fits are also in agreement with the findings of Guerrero and Di Federico^38^ in that the same value of attenuation may correspond to either a small size with a high COV or a larger, better-sorted sediment. Importantly, the range of fitted COVs is all larger than those measured via laser diffraction (Table 1) again inferring some potential errors from over shearing. The influence of the COV is also likely greater than found for the BPS, due to the calcite flocs larger sizes, resulting in both viscous and scattering attenuation.^32^

**Figure 8:**
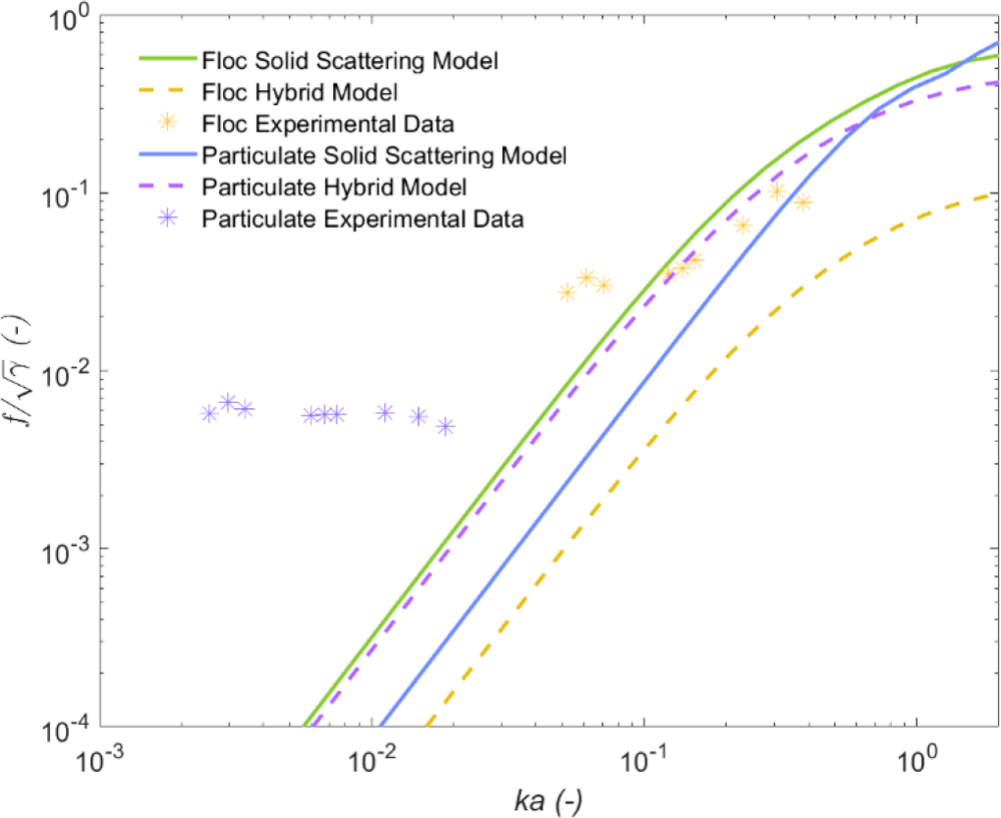
Form function for calcite flocs and particulates, normalized by √***γ***, compared to Solid Scattering and Hybrid models, as a function of frequency expressed in terms of *ka*.

To understand whether the Hybrid model could be further restricted or refined, form function data (taken from the experimental backscatter coefficients, *k*_*s*_) were investigated in a similar way with the flocculated calcite. The experimental form function data were normalized by the square root of the specific gravity (again following Bux et al.^39^) and are presented along with both Solid Scattering and Hybrid model predictions in Figure 8.

The particulate calcite data are very poorly fitted to the scattering models (unlike in the case of the attenuation values; see Figure 5). The experimental deviations from the modeled data are attributed here to the aforementioned lack of data sets in the low *ka* region used to fit the model^23,24^ that has been observed by previous authors.^39^ It emphasizes the need to extend heuristic modeling to account for the decreasing change in scattering cross-section and form function with frequency at low *ka*, observed in experimental data sets. The physical cause of this plateau is unknown, although it may correspond to multiple scattering effects increasing the noise in the system, thus artificially enhancing the backscatter coefficient, *k*_*s*_, and hence the form function, *f*. Alternatively, it may be the case that the COV is under-reported during size measurements that would otherwise cause a plateau in the form function at high and low *ka* values. Greater degrees of polydispersity have previously been noted to elevate backscatter form function values.^38,67^

The majority of the experimental data for the flocculated calcite lie between the Solid Scattering and Hybrid models. The flocculated calcite data at low frequencies are greater than those of the Solid Scattering model and exhibit a reduced sensitivity to frequency than predicted. This decreased sensitivity was also observed in the scattering cross-section results for flocculated calcite and may similarly indicate that, in modeling terms, a greater COV is measured by the ABS compared to the light scattering, when using the same mean number size. The form function data for flocculated calcite also support the scattering cross-section results, in that some degree of reduced scattering and attenuation is apparent for the flocs, compared to model data for a solid particle of the same size, in agreement with a number of previous results.^23,26,61,68,69^

**Figure 9:**
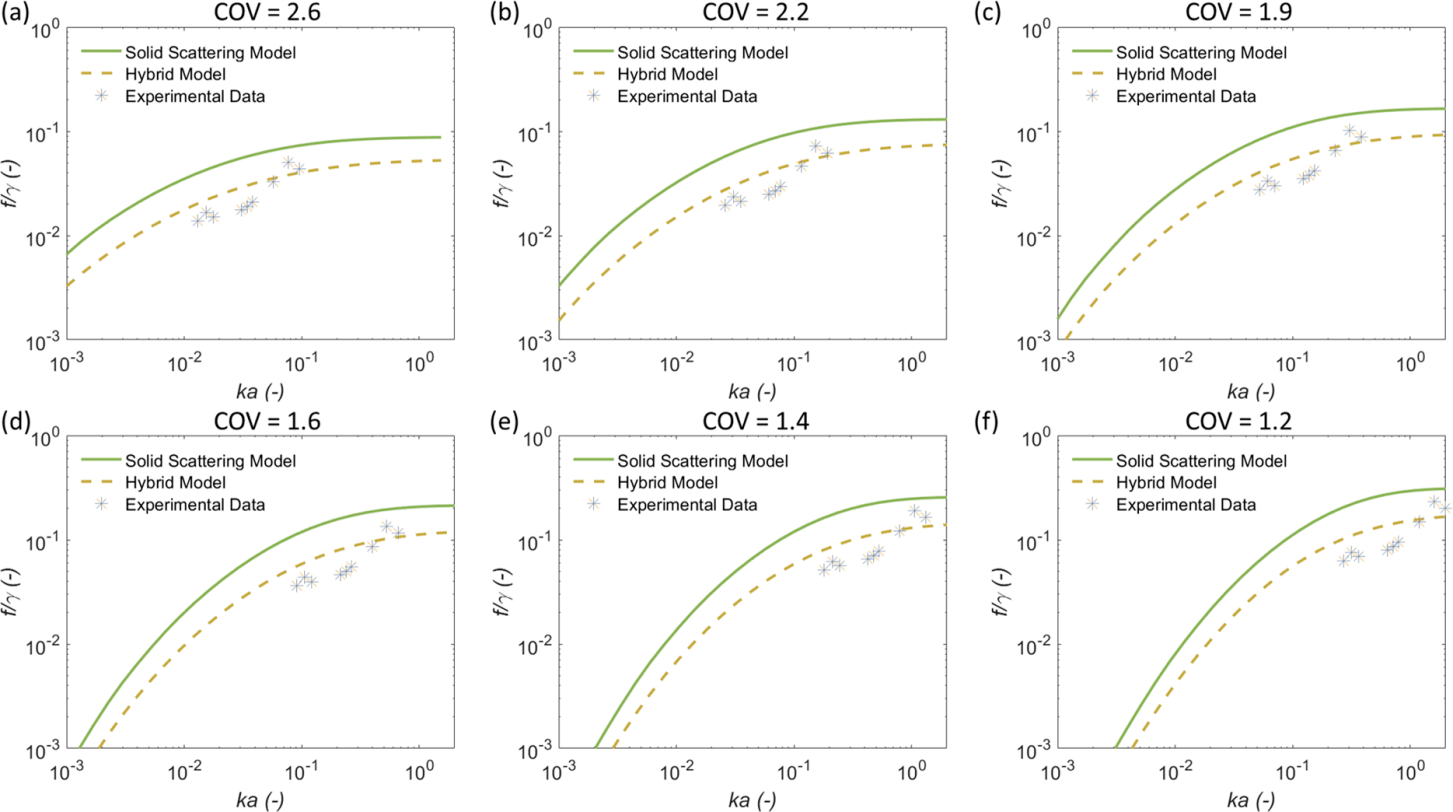
Comparison of experimental normalized form function data for calcite flocs with Solid Scattering and Hybrid models, using number mean sizes of a) 7.2, b) 14.5, c) 28.9, d) 50, e) 100, and f) 150 μm, with corresponding floc densities of 2531, 2281, 2195, 2131, 2055, and 2013 kg·m^–3^ and fitted COVs, respectively.

To compare to optimized fits of the scattering cross-section produced for the flocculated calcite data, modeling variables (mean aggregate size, fractal dimension, and the COV) were again fitted using the same procedure (with sizes varied at set levels and the COV used as a free fitting parameter). A set fractal dimension value of 2.9 was also found to give closest approximations, with results presented in Figure 9. While empirical fits to existing form function and scattering cross-section data have been produced to take account of a changing COV by Thorne and Meral,^24^ these are limited to values of *ka* above 0.1. While useful when accurate size data are limited, they are otherwise less rigorous than calculating the ensemble form function and scattering cross-section values directly.

Similar to the scattering cross-section results, the same degree of accuracy in model fits can be replicated using either a smaller mean size with a larger COV or a large size with a smaller COV. Therefore, there is limited additional confirmation from the form function fits when considering the parameter variation. However, with regards to eliminating potential outliers, it is not thought that the larger mean size fits ((d)–(f)) are reasonable, as they considerably differ from the size measured using light scattering (noting that similar sizes were measured from the *in situ* FBRM, Figure 1). Considering the likely floc sizes, data are best represented by (c), showing that fits can be considerably improved through an increase in the COV alone. Again, this suggests that the measured COV from light scattering seems to be under-reported for their true value. Nonetheless, as with the cross-section results, nearly identical fits can be obtained using different combinations of floc size and the COV. Therefore, some degree of *a priori* system knowledge or secondary measurement of size or polydispersity is still required to better constrict the model solutions.

It is interesting that both scattering cross-section and form function fits required an arbitrary high value of fractal dimension (*D*_*f*_*=* 2.9) that was considerably larger than measured using light scattering. The high floc density that is therefore measured by the acoustics may indicate that the fractal dimension measured using static light scattering via sampling may not adequately capture the shear breakdown of the flocs in the calibration column. While the *in situ* FBRM suggested only minor changes in size over time, it may be that constant shearing and reaggregation could density the flocs. Alternatively, as the experimental data for the flocculated particle systems lie closer to the solid scattering model (i.e., a fractal dimension of *D*_*f*_*=* 3.0), the theory proposed by MacDonald and co-workers^26,27^ may again hold, where the scattering is dominated by the tightly bound flocculi that make up the microstructure of the floc. Indeed, it may be the case in many systems that it is not actually critical to model aggregates as fractals, where differences to well dispersed solids may be more due to additional polydispersity in flocculated suspensions.

To further improve the model, it is suggested that viscous layer overlap effects must be included,^28^ coupled with a more thorough understanding of the changes in interparticle spacing during flocculation and shear breakdown. Elucidating the effects of floc microstructure versus macrostructure on ultrasonic scattering would enable greater accuracy in modeling the acoustic scattering cross-section of flocculated particle systems, ultimately giving more certainty in model fits without the need for independent measurements. Also, future work will look to assess alternative techniques to measure fractal dimensions along with acoustic analysis *in situ* (e.g., video probe microscopy) to enable more direct correlation.

### 4.3 Concentration Inversion Profiles

**Figure 10:**
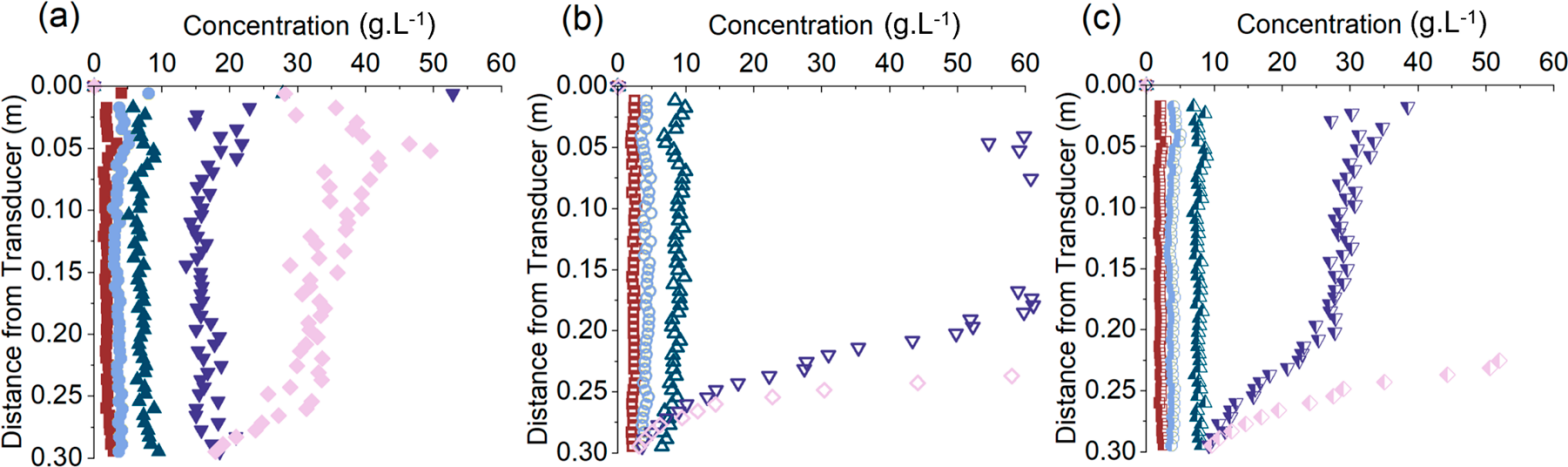
Dual-frequency inversion profiles for flocculated calcite at concentrations of ■ 2.3 g·L^–1^, ● 4.1 g·L^–1^, ▲ 8.3 g·L^–1^, ▼ 18.7 g·L^–1^, and ◆ 34.9 g·L^–1^ using frequency pairings of a) 0.85 and 2.25 MHz, b) 2 and 5 MHz, and c) 0.85 and 5 MHz.

To further examine the effectiveness and limitations of the technique for practical applications, the dual frequency concentration inversion model was applied for various frequency pairs using well mixed suspensions in the calibration tank. As the concentrations were the same within the measurement zone (see Figures S5–S7), any measured variation would be from iterative model deviations or measurement errors, giving a simple way of investigating the robustness of the inversions across the concentration range. Profiles for the flocculated calcite are presented in Figure 10 for concentration ranges from 2.3 to 34.9 g·L^–1^ and frequency pairs of 0.85 and 2 MHz, 2.25 and 5 MHz, and 0.85 and 5 MHz.

It is clear that the best performing frequency pair is 0.85 and 2.25 MHz, as it is able to profile the mean concentration throughout the whole range (although, there is some deviation at distances > 0.25 m for 34.9 g·L^–1^). The 2 and 5 MHz pair only measure accurately up to 8.3 g·L^–1^, with very large deviations above this level, while the 0.85 and 5 MHz pair gain reasonable profiles up to 18.7 g·L^–1^ (although with some over-estimation at this value). The main reason for the limitation is most likely the influence of interparticle scattering on attenuation, which is more prevalent for the higher frequencies and at larger distances. In fact, it is evident from the raw backscatter response (Figure 2) that the 5 MHz probe hits the instrument noise floor at the 34.9 g·L^–1^ concentration, while the 2 MHz is also close to the noise floor at this particle level. Also, the point at which interparticle scattering events will influence the attenuation will occur much below the noise floor of the instrument.^34^

Indeed, consulting d*G/*d*r* values (Figure 4) it can be observed that instabilities begin to occur above a d*G/*d*r* value of approximately −10 Np·m^–1^, which may provide a direct indicator of the limit for this concentration inversion method using the UARP. It is thought that this is not a result of mathematical error propagation through the profile but a real limit to the concentration inversion model’s assumption of negligible multiple scattering,^4^ even when grounding the value of the attenuation coefficient by taking measurements at multiple concentrations.^22,39^ Similar results have also been demonstrated in glass particle dispersions studied by the authors previously,^34^ although, in that case, the frequency range was limited to 2 and 2.5 MHz. Overall, a clear improvement in the dual frequency data is shown in comparison to the prior study^34^ by extending the pair frequency range and using a lower frequency that is less susceptible to multiple scattering effects.

**Figure 11:**
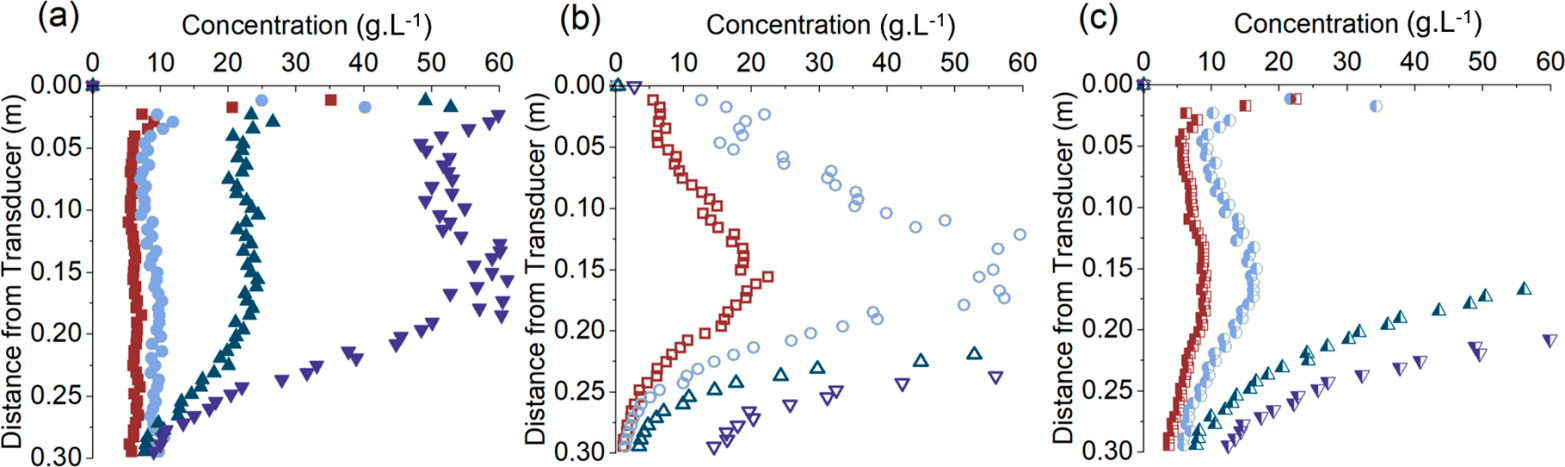
Dual-frequency inversion profiles for BPS at concentrations of ■ 6.9 g·L^–1^, ● 9.8 g·L^–1^, ▲ 16.7 g·L^–1^, and ▼ 21.2 g·L^–1^ with frequency pairings of a) 0.85 and 2.25 MHz, b) 2 and 5 MHz, and c) 0.85 and 5 MHz.

The BPS inversion data are shown in Figure 11, where broadly similar results were obtained. Nonetheless, for the two highest frequency pairs, in particular, the inversion becomes erroneous at lower concentrations than the calcite. Even for the low frequency pairing, the performance is reduced in comparison. These results are consistent with the raw backscatter response (Figure 3) where the instrument noise floor occurred at lower concentrations with the BPS than for the calcite, implying the higher levels of attenuation are reducing the operational concentration range (whether from the finer sediment causing more viscous attenuation or from greater levels of interparticle scattering).

The cause of the reduced profile limit between BPS and the flocculated calcite is interesting as the particle levels studied for each sediment are similar, demonstrating the impact of sediment properties on UARP inversion performance. Apart from the finer size, which will increase viscous attenuation and thus weaken the return signal for a given concentration, another difference separating BPS is the wider size distribution. It has been found previously by Salehi and Strom^70^ that, for signal-to-noise ratios below 30, individual calibrations across kaolinite sediments of different sizes and size distributions had to be performed to produce accurate inversion results. Intuitively, the conclusion may be drawn that an increase in size distribution may also lead to greater levels of attenuation and interparticle scattering and so also modify the noise floor of the ultrasonic system. Further, Vergne et al.^32^ considered the limitations of a number of inversion techniques and found that multiparameter inversions of both attenuation and scattering components may extend suitable sediment ranges. Nevertheless, it is emphasized that, at least for the lowest frequency pair, results indicate dual frequency profiling can be used at concentrations well above those normally studied in natural environmental sciences and at appropriate levels for monitoring the transfer and settling of nuclear sludge wastes,^2^ for example, giving high confidence in the technique.

## 5 Conclusions

In this study, backscattering and attenuation relationships were studied for two flocculated suspensions and a primary particle system using a bespoke high fidelity acoustic backscatter system (ABS) at multiple frequencies (1–5 MHz). Sediment specific attenuation (ξ) and backscatter (*k*_*s*_) coefficients were experimentally measured using the method of Bux et al.,^39^ and results compared to modeled values were calculated using the Hybrid model from Thorne et al.^23^ and the Solid Scattering model from Thorne and Meral.^24^ The sediment calibration procedure allowed for the determination of clear and robust estimates of ξ and *k*_*s*_ for all studied sediments. Nonflocculated particulate calcite fitted very closely to the Solid Scattering model for the dimensionless cross-section data, although over predicted scattering intensities, likely due to polydispersity effects. For the flocculated systems, experimental comparisons also fitted more closely to the Solid Scattering model than the Hybrid model, indicating that acoustic scattering may be dominated by tightly bound aggregates of primary particles that make up the microstructure of the floc. Nevertheless, by fitting the flocculated sediment data to the Hybrid model using the coefficient of variation (COV) with set particle sizes, it was possible to generate very good fits to both χ and *f*, with either smaller particle sizes and a high COV or larger more monodispersed suspensions. Thus, presently, some alternative size data are still required to bound model fit parameters. It is proposed here that viscous layer overlap may be the predominant cause of the reduction in attenuation for larger flocs, and future work developing a complete understanding of its effects may ultimately allow characterization without *a priori* knowledge of the sediment.

Additionally, dual-frequency inversions were used to generate concentration profiles of both flocculated sediments and best predicted using the lowest frequency pair (0.85 and 2 MHz) due to the lower interparticle scattering. Here, accurate concentrations up to 20–30 g·L^–1^ were possible, so long as d*G*/d*r* did not exceed ∼−10 Np·m^–1^. Such measurements have not been performed previously and represent a new advancement in the development of ABS as an *in situ* profiling device for cohesive sediments. The ability to measure higher concentrations increases the technique’s relevance for applications in engineering systems, where cohesive aggregated sediments are often encountered at intermediate concentrations during sludge settling and transport processes.

## Supporting Information Available

Fundamental backscatter voltage equations (eqs S1–S3) and Urick’s model for viscous adsorption (eqs S4–S7); particle size distributions for particulate and flocculated calcite (Figure S1) and BPS (Figure S2), as well as static light scattering data for fractal dimension measurements (Figure S3); measured transducer coefficients, *k*_*t*_ (Figure S4); calibration column concentration measurements (Figures S5–S7 and Table S1); measured *G*-function profiles for particulate calcite (Figure S8) and d*G*/d*r* versus *M* (Figure S9); measured attenuation coefficient values, ξ, (Table S2) and scattering coefficients, *k*_*s*_ (Table S3); modeled scattering cross-section with variations in fraction dimension (Figure S10), coefficient of variation (Figure S11), and compressional wave speed (Figure S12). T(PDF)
