# Supplementary material for: Characterizing Flocculated Mineral Sediments with Acoustic Backscatter, Using Solid and Hybrid Scattering Models
Source: Ind Eng Chem Res. 2023 Sep 27;62(42):17328–42. doi: 10.1021/acs.iecr.3c01874 (PMC10603782; doi:10.1021/acs.iecr.3c01874)
Supplement: Supplementary file 1 — ie3c01874_si_001.pdf [file ie3c01874_si_001.pdf]

# Supporting Information (SI)

## Characterizing flocculated mineral sediments with acoustic backscatter, using solid and hybrid scattering models

Alastair S. Tonge[a,b], Jeffrey Peakall[c], Alexander P.G. Lockwood[d], Martyn Barnes[d], Timothy N. Hunter[a*]

[a]*School of Chemical and Process Engineering, University of Leeds, Leeds LS2 9JT, UK.*

[b]*United Utilities Group PLC, Warrington WA5 3LP, UK.*

[c]*School of Earth and Environment, University of Leeds, Leeds LS2 9JT, UK.*

[d]*Sellafield Ltd, Hinton House, Birchwood Park Ave, Warrington WA3 6GR, UK.*

[*]Corresponding author: t.n.hunter@leeds.ac.uk

## S1. Fundamental backscatter voltage equations

The following model (Eq. S1) following Thorne and Hanes (2002), gives the variation of backscattered root-mean-square voltage, *V,* with distance from the transducer face, *r,* for a given mass concentration, *M,* of particles suspended in water for single particle scattering. Here, critically, $k_s$ is the particle species backscatter constant, while $\alpha_s$ is the attenuation constant for the sediment. Also, $\alpha_w$ is the attenuation due to water (which can be derived (Ainslie et al., 1998)) and $k_t$ is the transducer constant that captures the inherent gain of the system and probe characteristics. Additionally, $\psi$ is the near field correction factor (which tends to unity in the far field) and can be modelled as given by Downing et al. (1995), or as extended by the current authors (Tonge et al., 2021).

$$V(r) = \frac{k_t k_s M^{\frac{1}{2}}}{r\psi} e^{-2r(\alpha_w+\alpha_s)} \quad \text{S1}$$

The particle backscatter coefficient ($k_s$) can be related to the average particle radius ($a$) and solid density ($\rho_s$) through the dimensionless form function ($f$) as given in Eq. 2.

$$k_s = \frac{\langle f \rangle}{\sqrt{a\rho_s}} \quad \text{S2}$$

The particle attenuation constant can be related to the same parameters though the dimensionless scattering cross-section ($\chi$) using the mass independent sediment attenuation coefficient, $\xi$ (where $\alpha_s = \xi M$), in Eq. 3. In both (2) and (3), the angled brackets indicate a number average over the particle size distribution.

$$\xi = \frac{3\langle \chi \rangle}{4\langle a \rangle \rho_s} \quad \text{S3}$$

To relate the backscatter voltage to particle size and polydispersity or concentration properties, it is a requirement, therefore, to gain values of the backscatter and attenuation coefficients, either through direct measurements or modelling.

## S2. Urick's Model for Viscous Absorption

If viscous losses are to be accounted for when *ka << 1,* then the model of Urick (1948) can be used to calculate an additional attenuation term, $\chi_{\mathrm{sv}}$, caused by visco-inertial interactions between the particles and surrounding fluid. The additional viscous cross-section term (Eq. S4) is added on to the scattering cross section (Eq. S7). It is shown in terms of the density ratio between the spheres and the surrounding fluid ($\gamma$) and $\beta = \sqrt{\omega/2v}$, where $\omega$ is the acoustic angular frequency and *v* is the kinematic viscosity of water.

$$\chi_{\mathrm{sv}} = \frac{2}{3} x(\gamma-1)^2 \frac{\tau}{\tau^2 + (\gamma+\theta_v)^2} \quad \text{S4}$$

$$\tau = \frac{9}{4\beta a}(1+\frac{1}{\beta a}) \quad \text{S5}$$

$$\theta_v = \frac{1}{2}(1+\frac{9}{2\beta a}) \quad \text{S6}$$

$$\chi = \chi_{ss} + \chi_{sv} \quad \text{S7}$$

# S3. Supplementary Figures & Table data

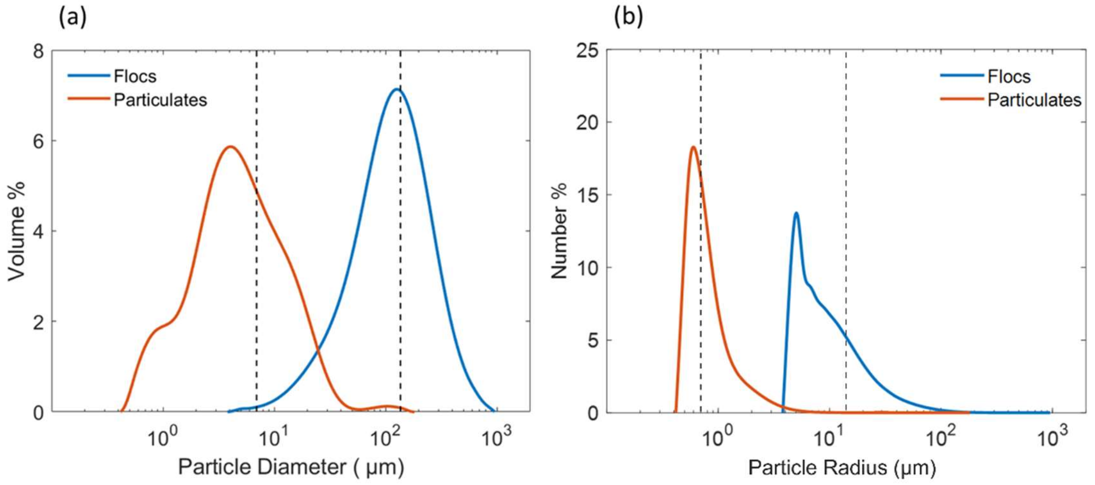


**Fig. S1: Particle size distributions (PSDs) for calcite particles and flocs, shown as a) volume distributions with dashed lines indicating the median size (*$d_{50}$*) and b) number distributions with dashed lines indicating the mean number radius (*$a_0$*).**

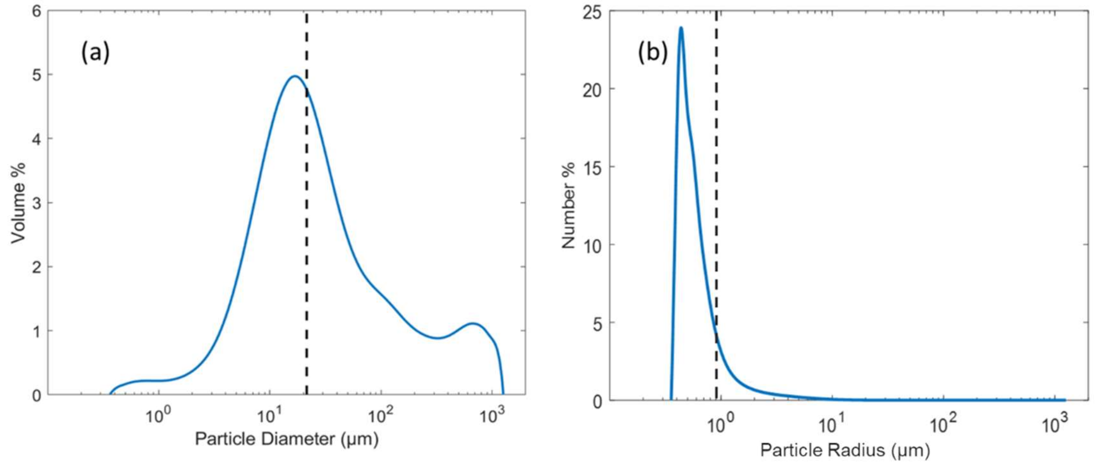


**Fig. S2: Particle size distributions for Barrnon pond sludge (BPS), shown as a) a volume distribution with dashed line indicating the *$d_{50}$* and b) a number distribution with dashed line indicating the mean number *$a_0$*.**

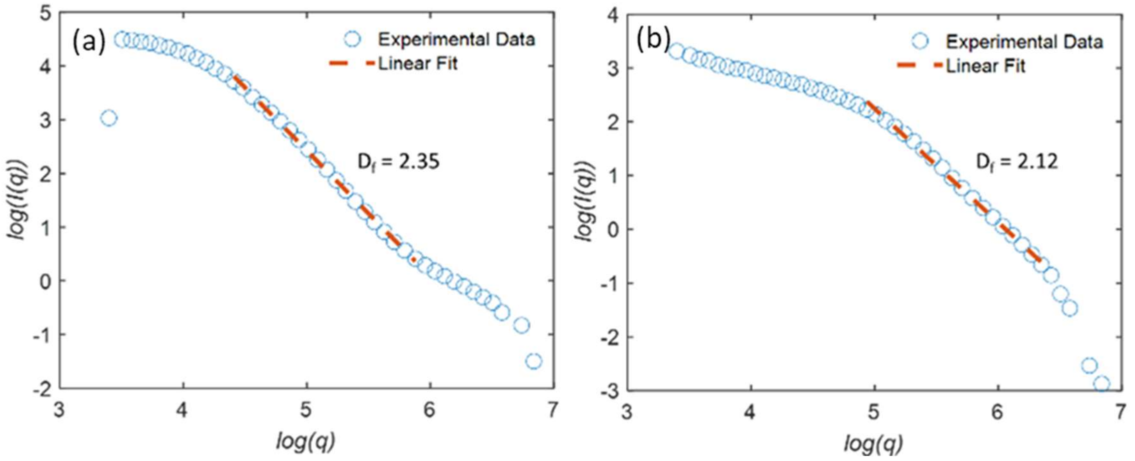


**Fig. S3: Light scattering data for a) flocculated calcite and b) BPS used to find the fractal dimension.**

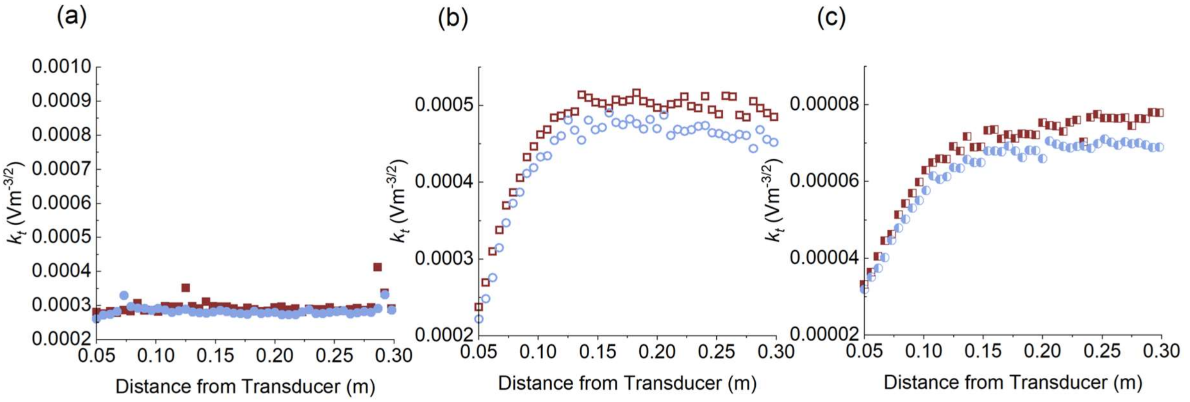


**Fig. S4: Transducer coefficient ($k_t$) calibration profiles at nominal concentrations of ■ 2.3 $g.L^{-1}$, ● 4.1 $g.L^{-1}$ for a) 1 MHz, b) 2.25 MHz and c) 5 MHz *Sonatest*$^{TM}$ probes operating at their central frequency, produced using *Honite 16*. Averaged values used for all calculations are taken from the far field (0.15–0.25 m).**

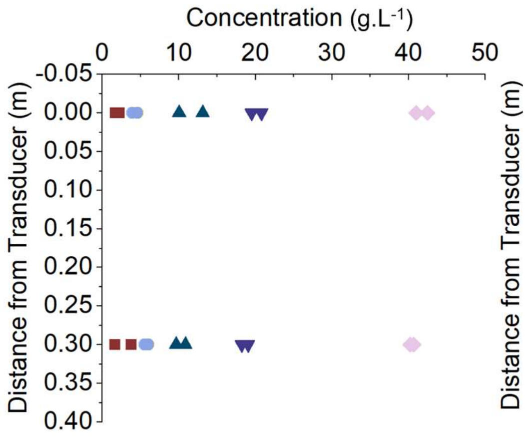


**Fig. S5: Gravimetric sample data for particulate calcite at nominal concentrations of ■ 2.3 g.L$^{-1}$, ● 4.1 g.L$^{-1}$, ▲ 8.3 g.L$^{-1}$, ▼ 18.7 g.L$^{-1}$, ◆ 34.9 g.L$^{-1}$.**

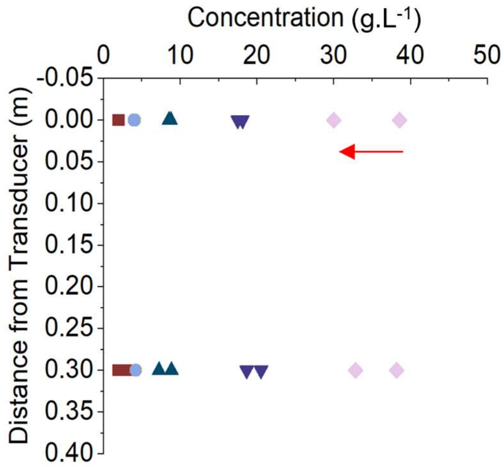


**Fig. S6: Gravimetric sample data for flocculated sediments calcite at nominal concentrations of ■ 2.3 g.L$^{-1}$, ● 4.1 g.L$^{-1}$, ▲ 8.3 g.L$^{-1}$, ▼ 18.7 g.L$^{-1}$, ◆ 34.9 g.L$^{-1}$. Red arrows indicate where a notable change in concentration was observed in samples before and after acoustic measurement.**

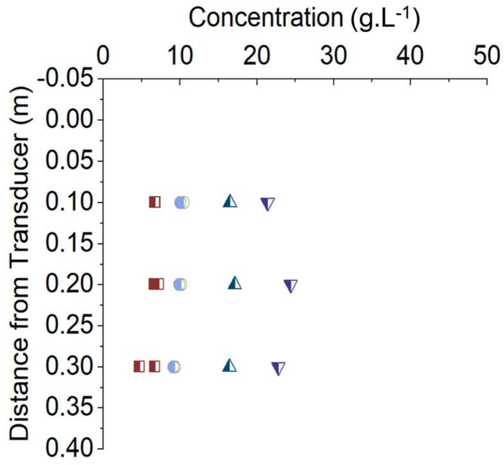


**Fig. S7: Gravimetric sample data for Barrnon Pond Sludge (BPS) at nominal concentrations of ■ 6.9 g.L$^{-1}$, ● 9.8 g.L$^{-1}$, ▲ 16.7 g.L$^{-1}$, ▼ 21.2 g.L$^{-1}$.**

**Table S1: Nominal weighed and measured concentrations for all cohesive sediment systems studied (all concentrations are in g.L$^{-1}$).**

| | Calcite Particulates | Calcite Flocs | Barrnon Pond Sludge | |
|---|---|---|---|---|
| Nominal | Measured | Measured | Nominal | Measured |
| 2.5 | 2.4 | 2.3 | 5.0 | 6.9 |
| 5.0 | 5.0 | 4.1 | 10.0 | 9.8 |
| 10.0 | 10.9 | 8.3 | 15.0 | 16.7 |
| 20.0 | 19.4 | 18.7 | 20.0 | 22.9 |
| 40.0 | 41.1 | 34.9 | - | |

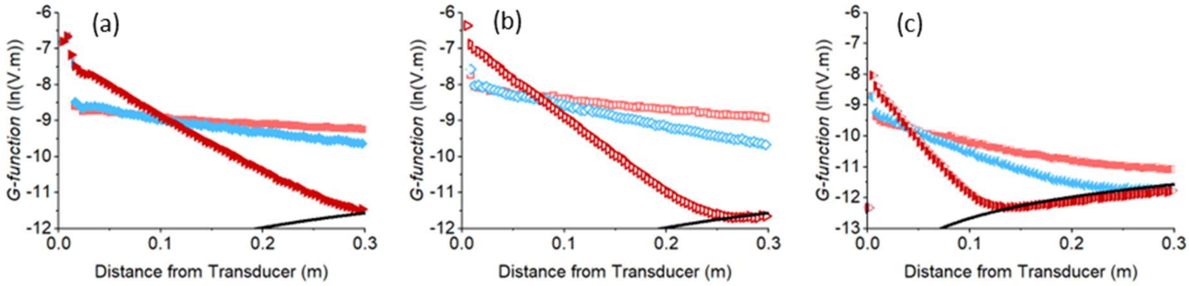


**Fig. S8: Measured *G*-function profiles ((a) – (c)) for non-flocculated particulate calcite pulsed at 1, 2.25 and 5 MHz respectively, from three particle concentrations (■ = 2.3 g.L$^{-1}$, ◆= 8.3 g.L$^{-1}$, ▶= 34.9 g.L$^{-1}$).**

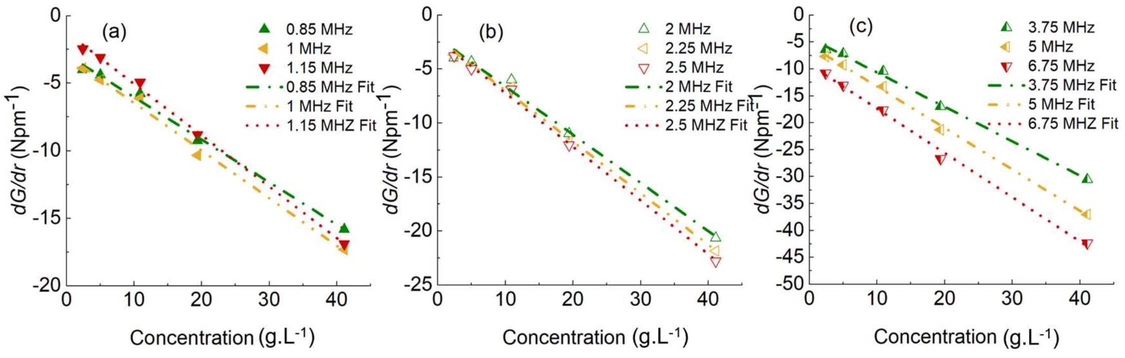


**Fig. S9: Gradient *dG/dr* versus concentration calibrations for particulate calcite to allow for determination of attenuation coefficient at a) 1, b) 2.25 and c) 5 MHz.**

**Table S2: Attenuation coefficient, $\xi$ ($Np.m^{-3}.g^{`1}.L^{-1}$), values with corresponding correlation $R^2$ values used for the fit.**

| | Calcite Particulates | | Calcite Flocs | | BPS | |
|---|---|---|---|---|---|---|
| | $\xi$ | $R^2$ | $\xi$ | $R^2$ | $\xi$ | $R^2$ |
| 0.85 MHz | 0.16 | 0.995 | 0.12 | 0.991 | 0.17 | 0.990 |
| 1 MHz | 0.18 | 0.992 | 0.14 | 0.989 | 0.19 | 0.996 |
| 1.15 MHz | 0.19 | 0.997 | 0.15 | 0.996 | 0.21 | 0.987 |
| 2 MHz | 0.22 | 0.994 | 0.22 | 0.978 | 0.38 | 0.945 |
| 2.25 MHz | 0.24 | 0.997 | 0.23 | 0.995 | 0.43 | 0.993 |
| 2.5 MHz | 0.25 | 0.998 | 0.26 | 0.996 | 0.49 | 0.998 |
| 3.75 MHz | 0.32 | 0.998 | 0.40 | 0.991 | 0.75 | 0.978 |
| 5 MHz | 0.39 | 0.996 | 0.51 | 0.994 | 0.90 | 0.993 |
| 6.25 MHz | 0.41 | 0.995 | 0.56 | 1.000 | 0.78 | 0.974 |

**Table S3: Scattering coefficient, $k_s$ ($V.m^{-3/2}$), values and corresponding standard deviation over the range of concentrations averaged to produce the mean value.**

| | | Calcite Particulates | | Calcite Flocs | | BPS | |
|---|---|---|---|---|---|---|---|
| | | $k_s$ | σ | $k_s$ | σ | $k_s$ | σ |
| 0.85 MHz | Conc 1 | 0.27 | 0.009 | 0.240 | 0.004 | 0.330 | 0.008 |
| | Conc 2 | 0.17 | 0.006 | 0.210 | 0.004 | 0.250 | 0.007 |
| | Conc 3 | 0.15 | 0.005 | 0.150 | 0.003 | 0.260 | 0.010 |
| | Conc 4 | 0.20 | 0.005 | 0.140 | 0.004 | 0.240 | 0.004 |
| | Conc 5 | 0.24 | 0.007 | 0.170 | 0.005 | - | - |
| 1 MHz | Conc 1 | 0.31 | 0.016 | 0.300 | 0.006 | 0.420 | 0.014 |
| | Conc 2 | 0.20 | 0.011 | 0.250 | 0.009 | 0.340 | 0.017 |
| | Conc 3 | 0.18 | 0.011 | 0.190 | 0.006 | 0.350 | 0.014 |
| | Conc 4 | 0.25 | 0.012 | 0.180 | 0.007 | 0.330 | 0.012 |
| | Conc 5 | 0.30 | 0.017 | 0.230 | 0.007 | - | - |
| 1.15 MHz | Conc 1 | 0.28 | 0.008 | 0.280 | 0.007 | 0.410 | 0.017 |
| | Conc 2 | 0.18 | 0.009 | 0.230 | 0.015 | 0.370 | 0.008 |
| | Conc 3 | 0.15 | 0.017 | 0.180 | 0.010 | 0.340 | 0.020 |
| | Conc 4 | 0.21 | 0.022 | 0.180 | 0.013 | 0.330 | 0.021 |
| | Conc 5 | 0.28 | 0.016 | 0.220 | 0.013 | - | - |
| 2 MHz | Conc 1 | 0.26 | 0.015 | 0.300 | 0.005 | 0.360 | 0.034 |
| | Conc 2 | 0.16 | 0.009 | 0.280 | 0.013 | 0.340 | 0.027 |
| | Conc 3 | 0.13 | 0.017 | 0.260 | 0.015 | 0.300 | 0.036 |
| | Conc 4 | 0.19 | 0.016 | 0.350 | 0.005 | 0.270 | 0.037 |
| | Conc 5 | 0.23 | 0.015 | 0.320 | 0.023 | - | - |
| 2.25 MHz | Conc 1 | 0.26 | 0.017 | 0.300 | 0.025 | 0.430 | 0.028 |
| | Conc 2 | 0.17 | 0.010 | 0.330 | 0.014 | 0.360 | 0.039 |
| | Conc 3 | 0.15 | 0.015 | 0.310 | 0.020 | 0.320 | 0.048 |
| | Conc 4 | 0.22 | 0.008 | 0.360 | 0.022 | 0.330 | 0.034 |
| | Conc 5 | 0.23 | 0.019 | 0.360 | 0.029 | - | - |
| 2.5 MHz | Conc 1 | 0.26 | 0.012 | 0.360 | 0.010 | 0.450 | 0.039 |
| | Conc 2 | 0.17 | 0.010 | 0.340 | 0.026 | 0.480 | 0.029 |
| | Conc 3 | 0.18 | 0.004 | 0.380 | 0.014 | 0.390 | 0.052 |
| | Conc 4 | 0.20 | 0.012 | 0.400 | 0.028 | 0.330 | 0.059 |
| | Conc 5 | 0.23 | 0.015 | 0.440 | 0.012 | - | - |
| 3.75 MHz | Conc 1 | 0.27 | 0.029 | 0.520 | 0.047 | 0.600 | 0.073 |
| | Conc 2 | 0.17 | 0.014 | 0.570 | 0.048 | 0.700 | 0.067 |
| | Conc 3 | 0.15 | 0.018 | 0.570 | 0.065 | 0.580 | 0.091 |
| | Conc 4 | 0.20 | 0.014 | 0.620 | 0.079 | 0.580 | 0.085 |
| | Conc 5 | 0.20 | 0.021 | 0.630 | 0.061 | - | - |
| 5 MHz | Conc 1 | 0.21 | 0.044 | 0.740 | 0.077 | 0.690 | 0.110 |
| | Conc 2 | 0.21 | 0.016 | 0.950 | 0.031 | 0.710 | 0.110 |
| | Conc 3 | 0.13 | 0.029 | 0.900 | 0.079 | 0.660 | 0.098 |
| | Conc 4 | 0.16 | 0.032 | 1.100 | 0.068 | 0.680 | 0.110 |
| | Conc 5 | 0.17 | 0.036 | 0.640 | 0.110 | - | - |
| 6.75 MHz | Conc 1 | 0.22 | 0.039 | 0.710 | 0.120 | 0.610 | 0.044 |
| | Conc 2 | 0.15 | 0.028 | 0.750 | 0.120 | 0.560 | 0.047 |
| | Conc 3 | 0.15 | 0.028 | 0.750 | 0.120 | 0.400 | 0.014 |
| | Conc 4 | 0.17 | 0.026 | 0.710 | 0.110 | 0.370 | 0.040 |
| | Conc 5 | 0.17 | 0.032 | 0.600 | 0.100 | 0.000 | 0.000 |

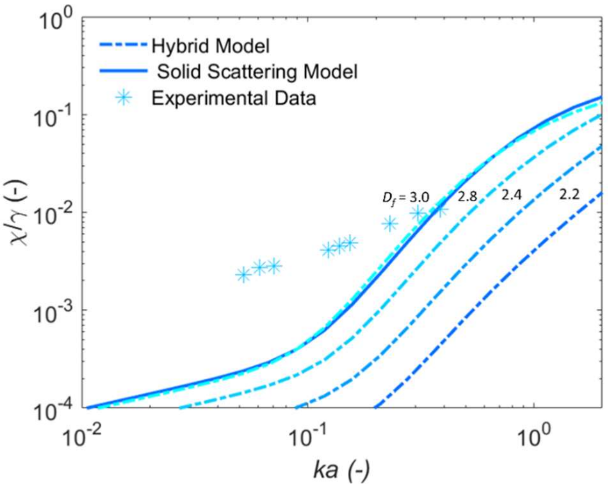


**Fig. S10: Specific gravity normalized scattering cross-section for flocculated calcite as a function of frequency expressed in terms of *ka,* with the effect of a variation in fractal dimension ($D_f$) shown for the Hybrid model.**

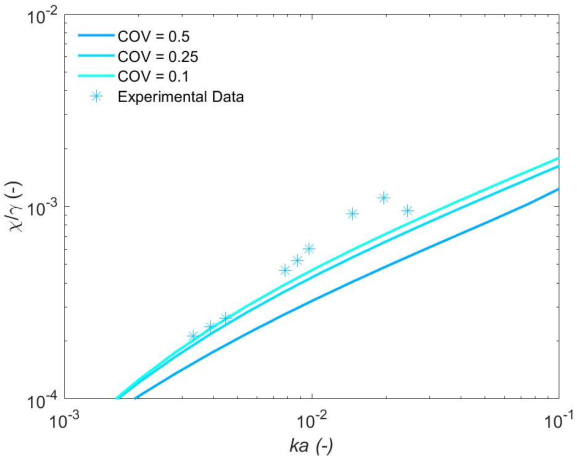


**Fig. S11: Specific gravity normalized scattering cross-section for BPS, as a function of frequency expressed in terms of *ka,* with the effect changing the coefficient of variation (COV) on the solid scattering model.**

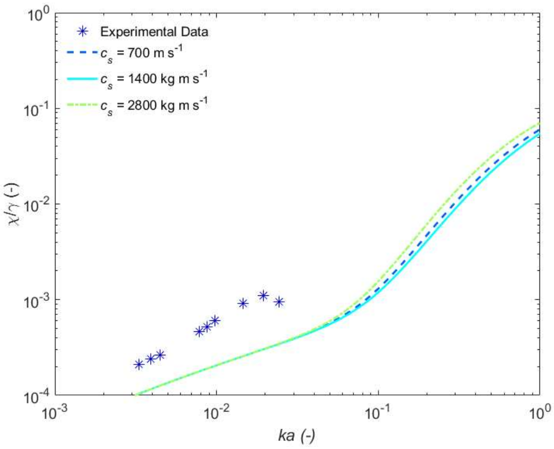


**Fig. S12: Specific gravity normalized scattering cross section for BPS as a function of frequency expressed in terms of *ka,* with the effect changing the compressional wave speed shown for the Hybrid model.**

## References (all also appear within the main manuscript in number format)


Ainslie, M. A.; McColm, J. G., A simplified formula for viscous and chemical absorption in sea water. *The Journal of the Acoustical Society of America* **1998,** *103*, 1671-1672.

Downing, A.; Thorne, P. D.; Vincent, C. E., Backscattering from a suspension in the near field of a piston transducer. *The Journal of the Acoustical Society of America* **1995,** *97*, 1614-1620.

Thorne, P. D.; Hanes, D. M., A review of acoustic measurement of small-scale sediment processes. *Continental Shelf Research* **2002,** *22*, 603-632.

Tonge, A. S.; Peakall, J.; Cowell, D. M. J.; Freear, S.; Barnes, M.; Hunter, T. N., Experimental validation of acoustic inversions for high concentration profiling of spherical particles, using broadband technology in the Rayleigh regime. *Applied Acoustics* **2021,** *180*, 108100.

Urick, R. J., The absorption of sound in suspensions of irregular particles. *The Journal of the Acoustical Society of America* **1948,** *20*, 283-289.
